# Flow-cytometric analysis of membrane integrity of stallion sperm in the face of agglutination: the “zombie sperm” dilemma

**DOI:** 10.1007/s10815-021-02134-z

**Published:** 2021-05-15

**Authors:** Isabel Ortiz, Matheus Felix, Hélène Resende, Luisa Ramírez-Agámez, Charles C. Love, Katrin Hinrichs

**Affiliations:** 1 College of Veterinary Medicine & Biomedical Sciences, Texas A&M University, College Station, TX USA; 2 Department of Clinical Studies-New Bolton Center, University of Pennsylvania, 382 W. Street Rd., Kennett Square, PA 19348 USA

**Keywords:** Stallion, Sperm, Acrosome, Membrane integrity, Flow cytometry, A23187

## Abstract

**Purpose:**

To define the effect of sperm agglutination, associated with incubation under capacitating conditions, on accuracy of membrane assessment via flow cytometry and to develop methods to mitigate that effect.

**Methods:**

Sperm motility was measured by CASA. Sperm were stained with PI-PSA or a novel method, LD-PSA, using fixable live/dead stain and cell dissociation treatment, before flow-cytometric analysis. Using LD-PSA, acrosome reaction and plasma membrane status were determined in equine sperm treated with 10 μm A23187 for 10 min, followed by 0, 1, or 2 h incubation in capacitating conditions.

**Results:**

Using PI-PSA, measured membrane integrity (MI; live sperm) was dramatically lower than was total motility (TMOT), indicating spurious results (“zombie sperm”). Sperm aggregates were largely of motile sperm. Loss of motility after A23187 treatment was associated with disaggregation and increased MI. On disaggregation using LD-PSA, MI rose, and MI then corresponded with TMOT. In equine sperm incubated after A23187 treatment, as the percentage of live acrosome-reacted sperm increased, TMOT decreased to near 0.

**Conclusion:**

Flow cytometry assesses only individualized sperm; thus, agglutination of viable sperm alters recorded membrane integrity. As viable sperm become immotile, they individualize; therefore, factors that decrease motility, such as A23187, result in increased measured MI. Disaggregation before assessment allows more accurate determination of sperm membrane status; in this case we documented a mismatch between motility and live acrosome-reacted equine sperm that may relate to the poor repeatability of A23187 treatment for equine IVF. These findings are of profound value to future studies on sperm capacitation.

**Supplementary Information:**

The online version contains supplementary material available at 10.1007/s10815-021-02134-z.

## Introduction

Conventional in vitro fertilization (IVF) is not successful in the horse [1]. Current work toward understanding the basis of equine IVF has been focused on factors affecting sperm capacitation in this species [2]. It is clear from multiple studies that exposure to the calcium ionophore A23187 can result in fairly consistent, but low, rates of fertilization [3–6]. While certain media components (notably, calcium, bovine serum albumin (BSA), and bicarbonate) are known to promote capacitation in other species [7, 8], the effect of these components on capacitation of equine sperm is unclear. For example, albumin is a cholesterol acceptor which promotes cholesterol efflux from the sperm plasma membrane -- an important mechanism in capacitation -- in other species including humans, pigs, and mice [9–11]. However, presence of BSA in the medium is not associated with a reduction in membrane cholesterol levels in equine sperm [12].

Evaluation of the effects of various medium components on capacitation-related changes in equine sperm, including induction of the acrosome reaction, could increase our understanding of factors affecting equine IVF. An important consideration in accurate assessment of membrane integrity and acrosome status is that incubation of sperm in “capacitation-type” media, i.e., media containing calcium, bicarbonate, and BSA, has been associated with pronounced head-to-head agglutination in equine sperm, as in sperm of other species [13–16]. In porcine sperm, this agglutination was found to be regulated by all three capacitation factors (calcium, bicarbonate, and albumin) and to be associated with the removal of an epididymal “anti-agglutinin” from the sperm membrane surface [17–19]. Subjective visual evaluation suggests that agglutinated sperm may largely represent motile and thus membrane-intact sperm [14, 20–22]. Agglutination becomes an issue during sperm evaluation as flow cytometry, the major technology utilized for high-throughput sperm analysis, analyzes only single cells. Thus, agglutinated sperm, which may represent a large proportion of the membrane-intact sperm in a population, may not be accurately evaluated by this method.

Two strategies could be applied to address the problem of agglutination in sperm assessed by flow cytometry: (1) preventing agglutination by finding substitutes for the factors responsible for agglutination; or (2) disaggregating the sperm after incubation, without altering the staining status of the cells, before the sperm are analyzed. Of these, the first approach would limit our ability to evaluate medium components known to be associated with capacitation, such as BSA. Thus, for assessment of the effect of capacitation-related components, developing a method for the second approach appears to be vital, that is, altering the procedures used for assessment to maximize the proportion of sperm in a sample that is included in the analysis.

Initially, the aim of this study was to evaluate the effects of calcium ionophore A23187 and BSA, alone or in combination, on the proportion of membrane intact (viable), acrosome-reacted equine sperm. After identifying problems associated with our initial analysis, we then focused on evaluating the effects of agglutination on measurement of these parameters. We developed a novel method to decrease agglutination in stained sperm before analysis, and thus increase the accuracy of assessment.

## Materials and methods

All of the procedures for these experiments were performed according to the *United States Government Principles for the Utilization and Care of Vertebrate Animals Used in Testing, Research and Training* and were approved by Laboratory Animal Care Committee at Texas A&M University (AUPs 2015-0026 and 2018-0032). All reagents were purchased from Sigma-Aldrich (St. Louis, MO, USA) unless otherwise stated.

### Animals and semen collection

Four healthy, sexually active American Quarter Horse stallions, 7 to 24 years old, were used as semen donors. Semen samples were collected using a Missouri-model artificial vagina (Missouri Model; Nasco, Ft. Atkinson, WI, USA) with an in-line nylon filter (Animal Reproduction Systems, Chino, CA, USA). Except where indicated, for each study, three ejaculates were collected from each of three stallions on separate days. Initial sperm membrane integrity (viability) was evaluated using the Nucleocounter (SP-100; Chemometec, A/S, Allerød, Denmark) as previously validated [23]. Only ejaculates having ≥ 70% membrane intact (viable) sperm were used.

### Sperm washing and treatment

#### Sperm washing

Immediately after collection, semen was extended 1:9 in Hanks’ balanced salt solution (HBSS; ThermoFisher Scientific, Waltham, MA, USA, #14025076), either with 7 mg/mL of essentially fatty-acid free bovine serum albumin (BSA; Sigma A3803; HBSS-BSA) designated P (with protein), or with no BSA (N; no protein), both adjusted to pH 7.4, according to the assigned condition (see “Experimental design”). The HBSS contains 4.17 mM sodium bicarbonate and 1.26 mM calcium chloride as supplied by the manufacturer. Extended semen was centrifuged at 400×*g* for 5 min, and the resultant pellet was resuspended to the same volume with the assigned medium and centrifuged again. The supernatant was removed, the pellet was resuspended in 1 mL of the assigned medium, and the concentration of the pellet was determined using the Nucleocounter. The pellet was resuspended with the assigned medium to a final concentration of 40 × 10^6^ sperm/mL.

#### Sperm treatment

The washed sperm suspension was divided into aliquots of 495 μL each. Aliquots were treated with calcium ionophore A23187 (A) or vehicle (V). For A23187 treatment, a primary stock solution of 10 mg/mL (19.1 mM) A23187 was prepared in DMSO, and aliquots stored at −20 °C. For use, an aliquot of the primary stock solution was thawed and diluted with HBSS to make a 1 mM A23187 2° stock solution. Vehicle was prepared using the same ratio of DMSO:HBSS as for the A23187 2° stock solution (5.24% DMSO). In the experiment in which the dosages of A23187 varied, the 1 mM 2° stock solution was diluted with vehicle to obtain working stock solutions of A23187 containing the same concentration of DMSO. Treatment was performed by adding 5 μL of A23187 2° or working stock solution, or 5 μL of vehicle, to a 495-μL sperm aliquot. The final concentration of DMSO in the sperm suspensions was 0.052%.

The treated sperm aliquots were exposed to the ionophore or vehicle at 37 °C in air for 10 min. Samples were then centrifuged at 400×*g* for 5 min, and resuspended in either 1 mL of LPB medium (HBSS supplemented with sodium lactate (38 mM), sodium pyruvate (1.17 mM), and 7 mg/mL fatty-acid free BSA; pH 7.4, adjusted to 300 mOsm with distilled water) or 1 mL INRA96 (IMV, L’Aigle, France), according to the experiment. The final sperm concentration was approximately 20 × 10^6^ sperm/mL. Sperm samples for evaluation were obtained immediately after resuspension (0 h) then the aliquots were incubated at 37 °C in air and assessed again after varying periods of incubation, as described below.

#### Sperm motility analysis

Sperm motility was analyzed by computer-assisted sperm analysis (CASA, IVOS II, Hamilton-Thorne, Beverly, MA, USA), as previously described by Salazar et al. [24]. Briefly, a 6-μL sample was assessed; 10 different fields were measured and at least 500 sperm were evaluated for each sample, at 45 frames/s at a frame rate of 60 Hz. Other settings were minimum contrast 70; minimum cell size 4 pixels; minimum static contrast 30; cell intensity 106 pixels; static head size 0.60–2.00 μM; static head intensity 0.20 to 2.01; static elongation 40 to 85; and illumination intensity 2200. Sperm were considered to be progressively motile if straightness (STR) was >50% and velocity of the average path (VAP) was >30 μm/s. The percentage of total (TMOT) sperm motility, percentage of progressive sperm motility (PMOT), and the average curvilinear velocity within the motile sperm (VCL, μm/s) were recorded.

#### Analysis of sperm plasma membrane and acrosome status

Sperm membrane integrity and acrosome status were assessed by flow cytometry using two different methods, the use of which varied among trials as outlined in the Experimental Design, as follows.

### PI-PSA method

Assessment via PI-PSA was performed using the method described by Salazar et al. [24] with minor modifications. Briefly, 40 μL of the selected sperm suspension was diluted in 133 μL of Dulbecco’s phosphate-buffered saline without calcium and magnesium (DPBS(--); Thermofisher). Then, 10 μL of a 62.5 μg/mL solution of FITC-conjugated *Pisum sativum* agglutinin (FITC-PSA) in DPBS(--) and 2 μL of a 2.4 mM solution of propidium iodide (PI) in distilled water were added, for final concentrations of 0.05 mg/mL PSA and 1.3 μM PI. The samples were then incubated at room temperature in the dark for 10 min. After this, 40 μL of the stained sample was added to 400 μL of DPBS(--) and this suspension was placed in the feed of the flow cytometer (FACScan, Becton Dickinson, Mountain View, CA, USA). The instrument was equipped with a 488-nm argon laser. The voltage settings were SSC 240, FSC 470, FL1 650, and FL2 657. The compensation was set for FL1 as 1.9% of FL2, and for FL2 as 47.2% of FL1. A minimum of 5000 total events was evaluated per sample. “Gating” (% of gated events) refers to the proportion of the total events that, based on forward scatter/side scatter, were considered to represent individual sperm, and thus were included in the analysis. Data were acquired using a log scale and analyzed by Winlist^TM^ software (Verity Software House, Topsham, ME, USA). Experimental endpoints analyzed were the percentages of membrane-intact sperm (MI), total acrosome-reacted (AR) sperm (tAR), and membrane intact, acrosome-reacted sperm (MI-AR).

### LD-PSA method

This procedure was developed after we obtained the results of the preliminary studies using the PI-PSA method. The results of those studies appeared to be inaccurate, as the proportion of MI sperm was notably lower than was TMOT; we attributed this to exclusion of agglutinated sperm on flow-cytometric analysis (see “Results” and “Discussion”). The goal of developing the new procedure was to increase the accuracy of evaluation of MI and AR in samples showing sperm agglutination. A stock solution of LIVE/DEAD^TM^ fixable red dead cell stain (LD; Thermofisher) was prepared by adding 50 μL of DMSO to the vial of LD powder, according to manufacturer’s recommendations. The LD stock solution was aliquoted and frozen at −20 °C. To perform staining, 50 μL of the selected sperm suspension was added to 950 μL of DPBS containing calcium and magnesium to obtain a sperm concentration of 1 × 10^6^ sperm/mL, then 1 μL of the LD stock solution was added. The sample was mixed and held at room temperature in the dark for 30 min. After this, 145 μL of 16% paraformaldehyde was added for fixation, to achieve a 2% final concentration of paraformaldehyde.

The fixed samples were stored at 4 °C in the dark until samples had been obtained for all timepoints in that replicate (2 to 6 h storage). The samples were then centrifuged, resuspended in 1 mL of DPBS (--) with 2 mg/mL non-fatty acid free BSA (Sigma A9647; DPBS(--)B), and centrifuged again. The pellet was suspended in 200 μL of Triton X, 1% in DPBS(--), to permeabilize the membranes. This mixture was held at room temperature in the dark for 10 min, then 800 μL of DPBS(--)B was added and the suspension centrifuged again. The supernatant was removed and the sperm pellet was resuspended in 133 μL of the cell dissociation solution, Accumax (Innovative Cell Technologies, Inc., San Diego, CA, USA). To this suspension, 10 μL of FITC-PSA was added (final concentration 0.0375 mg/mL) and the sample was held at room temperature in the dark for 10 min. This suspension was diluted with 200 μL of Accumax and the mixture was assessed by flow cytometry. The voltage settings for flow cytometry in this method were SSC 240, FSC 553, FL1 650, and FL2 657, and the compensation for this staining was set for FL1 as 0.0% of FL2, and for FL2 as 98.0% of FL1. Gating (% of gated events), MI, tAR, and MI-AR were recorded for analysis.

### Experimental design

#### Preliminary Study A: Use of the standard flow cytometry method (PI-PSA) to assess plasma membrane integrity and acrosome status in equine sperm treated with A23187 under various conditions

This preliminary study was performed using four ejaculates (one from each of four stallions). Aliquots of semen from each ejaculate were assigned to be washed, as described above, either in HBSS or in HBSS-BSA. Immediately after washing, sperm were exposed to 0 (V), 1 (C1), 5 (C5), or 10 (C10) μM A23187 for 10 min as described above. The sperm were then washed, resuspended in LPB medium, and incubated for 0, 0.5, 1, or 2 h. At each timepoint, samples of sperm suspension were removed for evaluation by CASA and for assessment by flow cytometry using the PI-PSA method.

#### Preliminary study B: Effect of anti-agglutinant measures (addition of D-penicillamine or use of milk-based extender) after A23187 exposure on sperm motility and on viability and acrosome status as assessed by the standard flow cytometry method (PI-PSA)

**Fig. 1:**
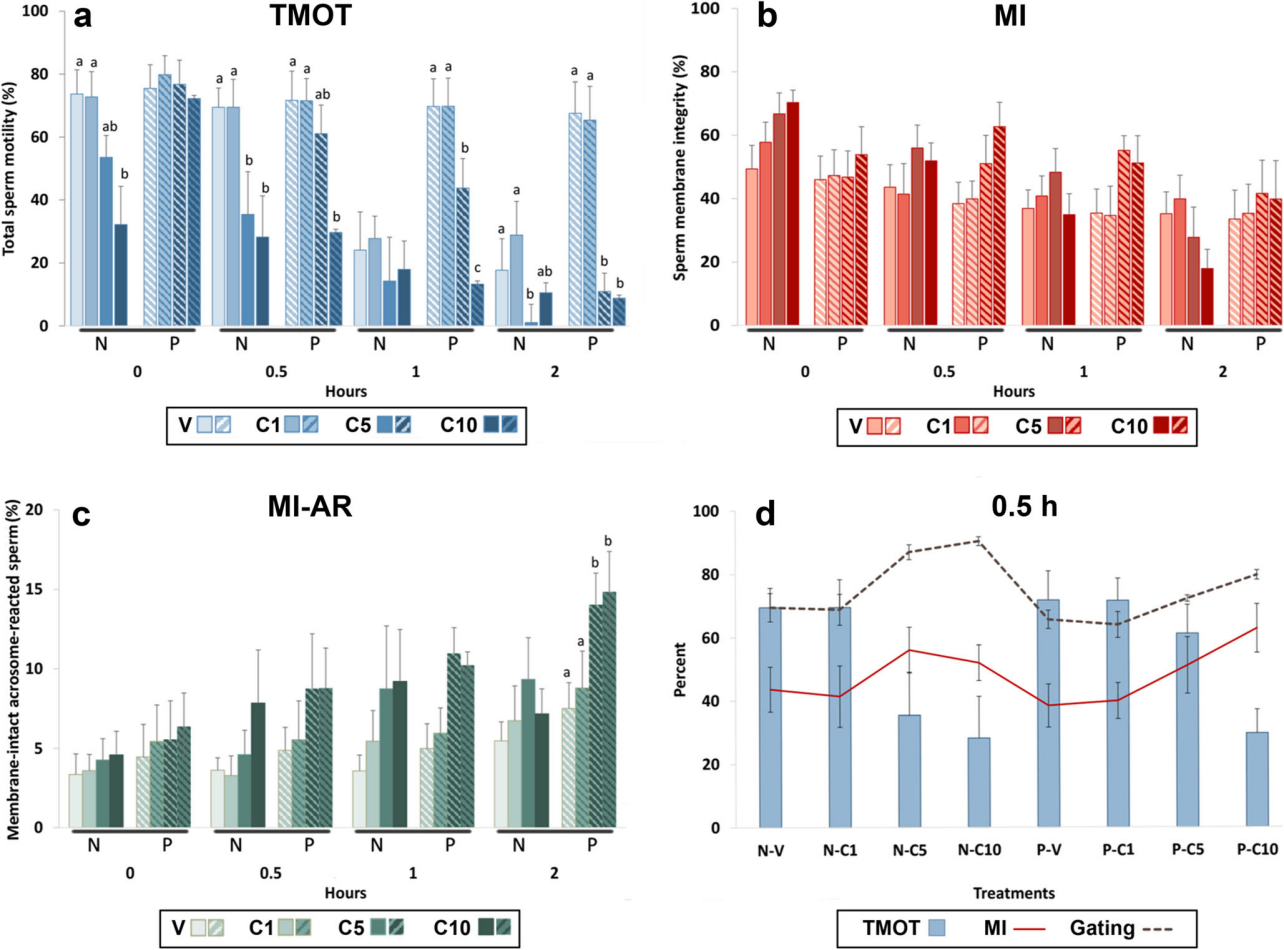
Effect of exposure of equine sperm to 0 (V), 1 (C1), 5 (C5), or 10 (C10) μM A23187 for 10 min in medium with (P) and without (N) 7 mg/mL bovine serum albumin, followed by washing and incubation for 0, 0.5, 1, or 2 h in LPB medium. Sperm total motility (TMOT,** a**) was assessed by CASA; membrane integrity (MI,** b**) and membrane intact-acrosome reacted sperm (MI-AR, **c**) were assessed by the PI-PSA method. Values are expressed as mean ± SEM. Within timepoint, different superscripts indicate significantly different values (*P* < 0.05). Panel **d** shows relationships among TMOT, Gating (percent of events included in flow cytometric analysis, i.e., individual non-agglutinated sperm), and MI after 0.5 h of incubation. Note that Gating was inversely related to TMOT, i.e., as TMOT decreased, the percentage of gated (individual) sperm increased. MI was parallel to Gating; i.e., as the Gating increased, the measured MI increased. In consequence, samples with lower TMOT had higher measured MI. Remarkably, for samples with high TMOT (V and C1 samples), the measured percentage of membrane-intact sperm was notably lower than was the percentage of total motile sperm

**Fig. 2:**
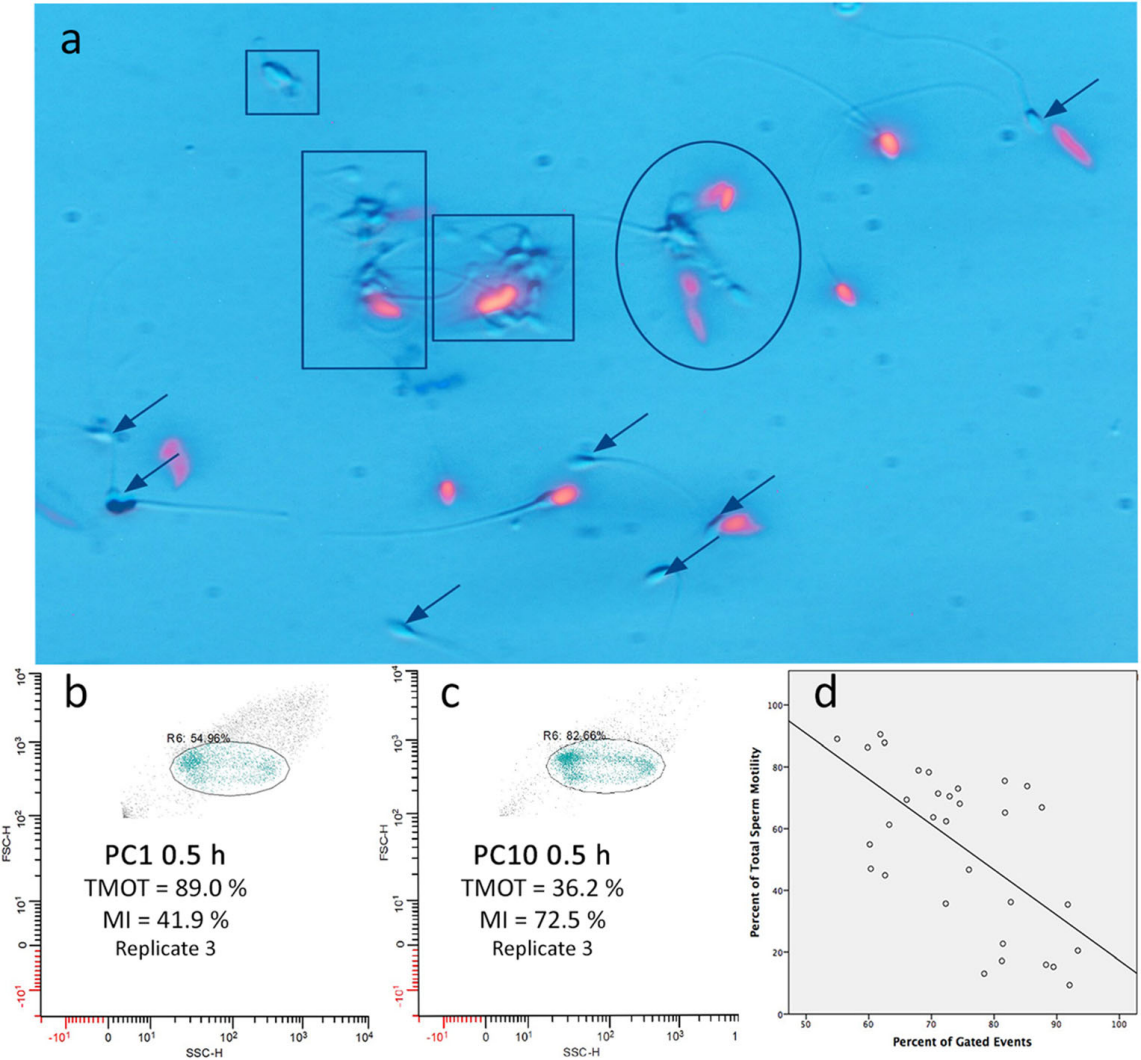
Interaction of equine sperm agglutination, Gating, and motility. **a**, Sperm stained with PI-PSA showing agglutinated (outlined) and individualized sperm, to help visualize the effect of agglutination on gating and measured percentage of membrane-intact (MI) sperm. Non-stained sperm are MI; stained sperm (red) are non-MI. Of 14 individualized sperm, 7 (arrows) were MI. In aggregates of agglutinated sperm, the proportions of MI/total sperm were the following (L to R): Small square, 3/3; rectangle, 9/11; large square, 14/17; oval, 6/10. Each outlined aggregate would likely be read by flow cytometry as one event, which, due to its greater side/forward scatter, would not be included in the gating. Thus, while overall, 39/55 (71%) of sperm in this entire field were MI, the flow cytometry measurement would include only the 14 individualized sperm and would thus report MI to be 7/14 (50%). The four aggregates would be counted as four events; thus, the total events for this field would be 14 individualized sperm + 4 aggregates, or 18 events. Gating would thus be reported as 14/18 (78%), whereas only 14/55 (25%) of the total sperm in the field were included in the flow cytometric analysis. (B, C) Gating profiles for samples from the same ejaculate, evaluated at 0.5 h of incubation after exposure to 1 (**b**) or 10 (**c**) μM A23187 for 10 min, showing the inverse relationship between TMOT and Gating. Total motility for the samples was 89% and 36%, respectively; Gating was 55% and 83%, respectively. **d**, Graph of linear regression showing the correlation between TMOT and Gating for all treatments (all replicates) at 0.5 h

In Preliminary Study A, we found that in some treatments, the proportion of MI sperm as measured by PI-PSA was markedly lower than was the total motility as measured by CASA (e.g., 46% MI sperm, with 76% TMOT for P-Vehicle at 0 h; see “Results,” Fig. 1). As for all mammalian sperm, motility of stallion sperm requires membrane integrity [25]; thus, the proportion of membrane-intact sperm should be equal to or exceed the proportion of motile sperm. The results obtained (a lower proportion of MI sperm than total motile sperm) implied that the data generated using the PI-PSA method were inaccurate. To explore the reason for this, we observed that after incubation in LPB, which contains albumin, the sperm were notably agglutinated on visual microscopic evaluation, as previously reported for equine and ovine sperm incubated in albumin-containing media [13, 14, 16]. Additionally, on CASA, we observed that samples with high motility tended to have sperm aggregates; in contrast, samples with low or no motility consisted almost entirely of individualized sperm. Thus, we hypothesized that viable, motile sperm incubated in the presence of albumin tended to agglutinate, and that as sperm lost motility, they disaggregated and became individualized. This hypothesis was supported by finding a significant negative correlation between both TMOT and VCL (as measured by CASA) and the Gating (i.e., events representing individual sperm) as measured on flow cytometry; i.e., as motility (TMOT or VCL) decreased, Gating increased (Results; Fig. 2d). This hypothesis was also supported by finding on visual microscopic evaluation of sperm stained with PI-PSA that the proportion of MI sperm appeared to be higher in sperm aggregates than in individualized sperm (Fig. 2a). We attributed the apparently spuriously low % of MI sperm in high-motility samples to the gating-out of sperm aggregates, which contained a large proportion of the MI sperm in the sample. This was supported by the paradoxical finding that at time 0, increasing dose of A23187 was associated with an increase in the reported percentage of MI (viable) sperm, while the sperm motility decreased (Fig. 1). Thus, we concluded that since motile viable sperm aggregated, in samples having high motility the population of sperm that were present as individualized sperm was biased toward being membrane-damaged (non-viable).

Preliminary study B was performed to evaluate the effects of two potential anti-agglutination measures: addition of D-penicillamine, which is reported to reduce agglutination in ovine sperm incubated in albumin-containing medium [14], or incubation in the commercial milk-based equine semen extender, INRA96 (IMV Technologies, L’Aigle, France; IN). One ejaculate from each of three stallions was used. Sperm were washed in HBSS. Immediately after washing, sperm were exposed to 0 (V) or 10 (C10) μM A23187 for 10 min. The sperm suspensions were then centrifuged and the pellet resuspended in one of three media: LPB, LPB containing 1 mM D-penicillamine (Millipore Sigma, St Louis, MO, USA; PEN) or IN. The suspensions were incubated for 0, 1, or 2 h. At each timepoint, samples were removed for evaluation by CASA and for assessment using the PI-PSA method.

**Fig. 3:**
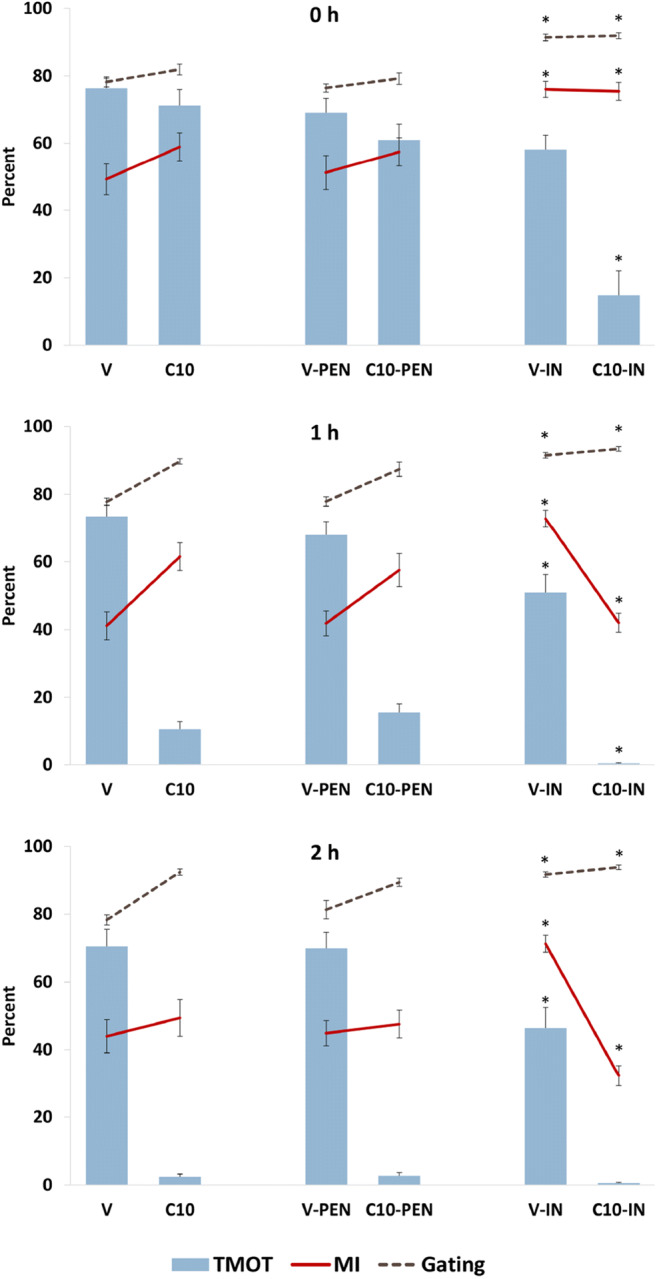
Effect of anti-agglutinant measures (1 mM D-penicillamine (PEN) or milk-based extender (IN)) in sperm exposed to 0 (V) or 10 (C10) μM A23187 for 10 min, followed by washing and incubation for 0, 1, or 2 h on total equine sperm motility (TMOT) assessed by CASA and on measured membrane integrity (MI) and Gating (percentage of gated events, i.e., individualized/non-agglutinated sperm) as assessed by the PI-PSA method. Asterisks denote significant differences from control

We noted that A23187-treated sperm that were subsequently resuspended in IN underwent an immediate, drastic reduction in motility, but not viability (see “Results,” Fig. 3). We hypothesized that this might be due to excess calcium influx in the sperm associated with residual amounts of A23187 interacting with a high calcium content in this milk-based extender. To explore this, we submitted three separate samples of each of the three media to the Clinical Pathology Laboratory at Texas A&M University for measurement of total (Vitros 4600, Ortho Clinical Diagnostics, Raritan, NJ, USA) and ionized (Stat Profile pHOx Ultra, Nova biomedical, Waltham, MA, USA) calcium.

#### Experiment 1: Comparison of measured sperm parameters as assessed by PI-PSA vs. LD-PSA methods

The results of the above preliminary studies formed a basis for our hypothesis on the interactions of agglutination and motility as they affect flow-cytometric measurement of membrane parameters. We then proceeded to focus on developing a method to mitigate the artifactual effect of the agglutination associated with incubation of sperm in capacitating conditions, i.e., in medium with calcium, bicarbonate, and notably BSA, on measured parameters.

Over a series of trials (data not shown), we developed a technique for membrane and acrosome staining which then allowed post-staining treatment to reduce agglutination before assessment by flow cytometry. This new protocol incorporated two anti-agglutination measures: treatment with Triton-X and the dissociating solution, Accumax. Since these treatments caused membrane damage in live sperm, we utilized a fixable live/dead stain (LD) that was compatible with sperm fixation with paraformaldehyde after staining and thus allowed subsequent post-fixation anti-agglutination treatment.

Experiment 1 was conducted to compare reported values, as assessed using the PI-PSA method vs. the LD-PSA method, for sperm membrane integrity and acrosome status after A23187 treatment followed by incubation in capacitating conditions.

Sperm from 9 ejaculates (3 ejaculates from each of 3 stallions) were washed with HBSS and treated with either vehicle (V) or 10 μM A23187 (C10) for 10 min, then centrifuged and resuspended in LPB. The suspensions were incubated for 0, 1, or 2 h. At each timepoint, samples were removed for evaluation by CASA and for assessment with both PI-PSA and LD-PSA methods. The effect of A23187 treatment on motility as measured by CASA, and differences between staining methods in the reported percentages of gated events, MI, tAR, and MI-AR as measured on flow cytometry, were compared for each A23187 treatment and timepoint.

#### Experiment 2: Comparison of results with the LD-PSA anti-agglutination method under various protocol modifications, including storage for 2 days before assessment

As the LD-PSA method is newly developed, this experiment was conducted to evaluate factors that might affect the results obtained using this method, including (A) initial semen resuspension and washing in HBSS vs. HBSS-BSA; (B) removal of stain/fixative by centrifugation before storage, which might allow a longer storage time; and (C) increasing the duration of storage up from ≤ 6 h to 48 h. The values for the measured parameters MI, tAR, and MI-AR were compared for sperm under two conditions: Vehicle at 0 h and 10 μM A23187 at 2 h.

**Fig. 4:**
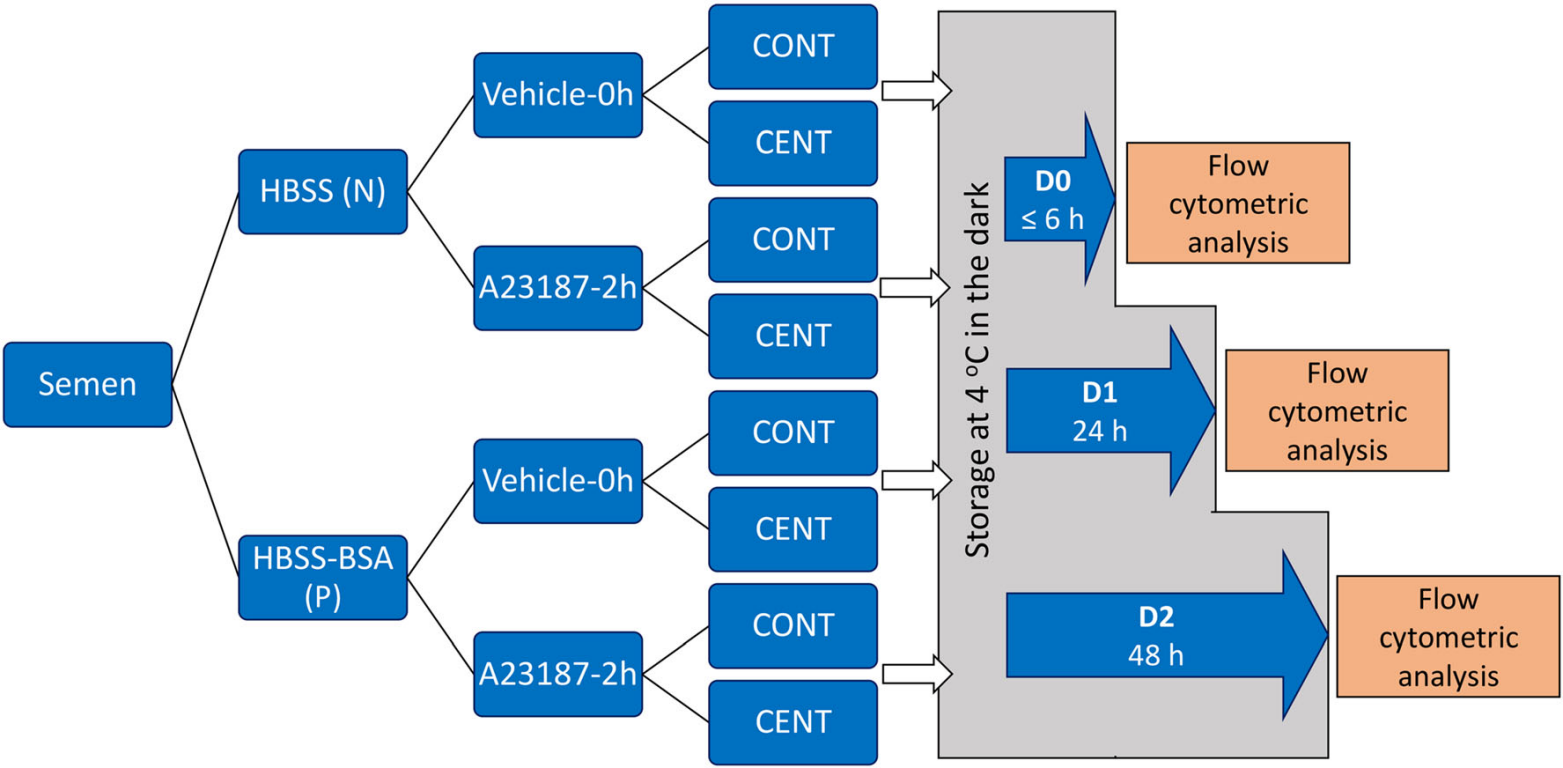
Flow chart showing the experimental design for Exp. 2. Sperm were washed in Hanks’ balanced salt solution (HBSS) with (P) or without (N) 7 mg/mL bovine serum albumin (BSA). Sperm were then exposed to 0 (vehicle) or 10 μM A23187 for 10 min, then washed and resuspended in LPB medium. Samples were incubated for 0 h (vehicle) or 2 h (A23187), before being stained with Live/Dead stain and fixed with paraformaldehyde. Samples were processed following the control (CONT, immediately placed into storage) or the CENT (centrifuged prior to storage) modification of the LD-PSA method. Samples were stored for ≤ 6 h (D0), 24 h (D1), or 48 h (D2) then processed as for the LD-PSA method. Plasma and acrosomal membrane parameters were compared among storage times (D0 vs. D1 vs. D2) within protocol modification (CONT, CENT)

Sperm from 9 ejaculates (3 ejaculates from each of 3 stallions) were washed, then treated with 0 (V) or 10 (C10) μM A23187. Sperm were resuspended in LPB and incubated for 0 or 2 h. Sperm were stained by the LD-PSA method as described above (control), or by the LD-PSA method with protocol modifications in a cascading design (Fig. 4), as follows:

A) Washing was performed in either HBSS (control, N) or HBSS-BSA media (protein-containing, P);
B) Sperm were treated with Vehicle or 10 μM A23187 for 10 min, then resuspended in LPB. Vehicle-treated sperm were stained at 0 h; A23187-treated sperm were stained after 2 h incubation.
C) After LD staining and addition of paraformaldehyde, samples were assigned to control centrifugation (CONT), i.e., two centrifugations after refrigerated storage; or split centrifugation (CENT), i.e., the first post-staining centrifugation and resuspension in DPBS(--)B was performed before placement into refrigerated storage, and the second centrifugation after removal from storage; and
D) To assess the effect of time in storage, each of the 8 conditions resulting from the above procedures was prepared in triplicate. Individual samples were analyzed after storage at 4 °C in the dark for ≤ 6 h (D0), 24 h (D1), or 48 h (D2), resulting in 24 treatments. Samples were analyzed by flow cytometry as described for LD-PSA staining, above. Results for MI, tAR, and MI-AR were compared within A23187 treatment between D0 and subsequent days among the protocol modification groups.

### Statistical analysis

Normality of the data distributions was evaluated using the Kolmogrov-Smirnov test. Because the data showed equal variance, comparisons among treatments over time were analyzed by repeated measures ANOVA with Greenhouse-Geisser and Bonferroni corrections. Pairwise comparisons were made with paired *T*-tests. Correlation between variables was performed using the Pearson correlation. The results were expressed as the mean ± standard error of the mean (s.e.). Results of ANOVA for the different protocols in Exps. 1 and 2 were validated using Bland-Altman plots to analyze the agreement between parameters. The differences between paired measurements were calculated, and the mean of the differences (*d*) was used to estimate the average bias of one method relative to the other. The 95% limit of agreement was calculated as *d* ± 1.96 SD, where SD is the standard deviation of the differences between paired measurements [26]. All statistical analyses were performed using SPSS version 20 (SPSS Inc, Chicago, IL, USA) and GraphPad Prism version 6 (GraphPad Software, San Diego, CA, USA) for Mac. The level of significance was set at *P* < 0.05.

## Results

### Preliminary study A: Effect of A23187 concentration, in medium with or without BSA, on sperm motility, plasma membrane integrity, and acrosome status as assessed by PI-PSA staining

The exposure of equine sperm to concentrations of calcium ionophore A23187 ≥ 5 μM significantly altered motility, as well as acrosomal membrane status and membrane integrity as analyzed by the PI-PSA method, in comparison to that for vehicle-treated controls (Fig. 1; suppl. Table 1).

Total motility (TMOT) was significantly decreased (*P* < 0.05) at 0 h when sperm were exposed to concentrations of A23187 ≥ 5 μM in N medium (without BSA), and at 0.5 h when sperm were exposed to ≥ 5 μM A23187 in P (BSA-containing) medium (Fig. 1a). Progressive motility (PMOT) followed the same pattern as did TMOT (suppl. Table 1). For sperm that were both membrane-intact and acrosome-reacted (MI-AR), significant differences from vehicle (*P* < 0.05) were found only at 2 h of incubation, for A23187 concentrations ≥ 5 μM in P (Fig. 1b). For total acrosome reaction (tAR), significant differences from vehicle (*P* < 0.05) were found at 2 h for A23187 concentrations ≥ 5 μM for both N and P (suppl. Table 1).

Unexpectedly, the measured percentage of MI sperm was significantly higher in samples treated with higher concentrations of A23187 than in vehicle-treated controls in N medium at 0 h and in P medium at 0.5 and 1 h (Fig. 1c). At these timepoints, extraordinarily, in the lower A23187 concentrations (vehicle and 1 μM A23187 treatments), the measured MI, indicating the percentage of viable sperm, was markedly lower than was the total motility as measured by CASA (data for 0.5 h presented in Fig. 1d). As motility *requires* membrane integrity, the higher motility than measured MI was incongruous. Motility measurement on the CASA is a well-validated technique and is visually confirmed during each analysis; therefore, given both this incongruity and the increase in measured MI with increasing dose of A23187, we suspected that the MI measurements as obtained by the PI-PSA method were spurious.

To evaluate causes for this suspected inaccuracy, we examined the gating profile of the samples as generated by flow cytometry. This parameter, Gating, reflected the % of gated events, i.e., those included in the analysis. The Gating included only the events within the forward scatter/side scatter (FSC/SSC) parameters established for individual sperm, and thus excluded events representing clumps of agglutinated sperm. At 0, 0.5, and 1 h, as TMOT decreased due to loss of motility in A23187-treated sperm, Gating increased, indicating that as motility was lost, sperm became individualized. Reported MI increased in parallel with Gating in these A23187-treated samples, i.e., as the percentage of gated (individual) sperm increased, the measured MI increased. This suggested that the non-motile sperm in A23187 treatments were largely still membrane-intact. Thus, both MI and Gating were inversely related to TMOT (shown at 0.5 h in Fig. 1d). We interpreted this to reflect that in samples incubated in capacitating-like conditions (presence of BSA, bicarbonate and calcium) such as the LPB medium in which all sperm were resuspended after A23187 or vehicle treatment in this study, viable motile sperm tend to agglutinate whereas non-motile sperm become individualized. In high-motility samples such as seen in the vehicle treatment, the non-motile, individualized sperm population is thus enriched for non-viable (non-membrane-intact) sperm. This hypothesis was supported by finding on microscopic evaluation of sperm stained with PI-PSA that the proportion of MI sperm appeared to be higher in sperm aggregates than in individualized sperm (Fig. 2a). For this reason, the MI reported on flow cytometry has a lower value than would be expected for that sperm sample, based on its motility. As motility decreased, especially in A23187-treated sperm, the percentage of sperm included in the gating (i.e., the proportion of individual sperm in the sample) increased (Fig. 2b,c). A negative correlation was found between TMOT and Gating at each timepoint (0.5 h shown in Fig. 2d) and for all timepoints combined (*r* = −0.608, *P* < 0.001). Similar results were found for VCL and Gating (*r* = −0.538, *P* < 0.01). This suggested that as sperm lost motility, they disaggregated and became individualized, and thus were included in the gating for flow cytometric analysis.

### Preliminary study B. Effect of anti-agglutinant measures (D-penicillamine or milk-based extender) after A23187 exposure on sperm motility, membrane integrity, and acrosomal status as assessed by the PI-PSA method

There were no significant differences (*P* > 0.05) in any evaluated sperm parameter at any time between samples resuspended in LPB and samples resuspended in LPB with 1 mM D-penicillamine (Fig. 3). In contrast, for samples resuspended in IN, Gating was significantly higher than in the LPB control for vehicle-treated samples at all timepoints (Fig. 3), indicating that there was a higher proportion of individualized sperm (i.e., less agglutination) in the vehicle-treated IN samples than in the vehicle-treated samples resuspended in LPB. In agreement with the hypothesis that in samples with low agglutination, individualized sperm (those analyzed by the flow cytometer) are more likely to be membrane intact, for vehicle-treated samples, the measured MI was significantly higher in samples resuspended in IN than in those resuspended in LPB. In agreement with the hypothesis that in non-agglutinated sperm, measured MI will be higher than TMOT, for IN samples the MI was higher than was the TMOT for V at all time periods. In agreement with the hypothesis that in agglutinated samples, as sperm lose motility they disaggregate and individualize, in LPB-suspended sperm, Gating and MI increased as motility decreased over time in A23187-treated sperm. The complete analysis is presented in Suppl. Table 2.

We noted that sperm resuspended in milk-based extender after A23187 treatment (C10-IN) showed significantly lower TMOT and PMOT (*P* < 0.001) than did controls. To explore the cause for this, we evaluated the calcium content of the different media. The values for total and ionized calcium were 1.2 ± 0.01 and 1.2 ± 0.01 mM, respectively, for HBSS; 1.1 ± 0.0 and 0.9 ± 0.0 mM, respectively, for LPB; and > 7 (above the standards of the instrument) and 1.8 ± 0.01 mM for IN. In IN medium, the measured MI became lower in the C10-IN treatment than in V-IN at 1 and 2 h, possibly reflecting sperm death due to prolonged excess intracellular calcium.

### Experiment 1: Comparison of measured sperm parameters as assessed by PI-PSA vs. LD-PSA methods

**Fig. 5:**
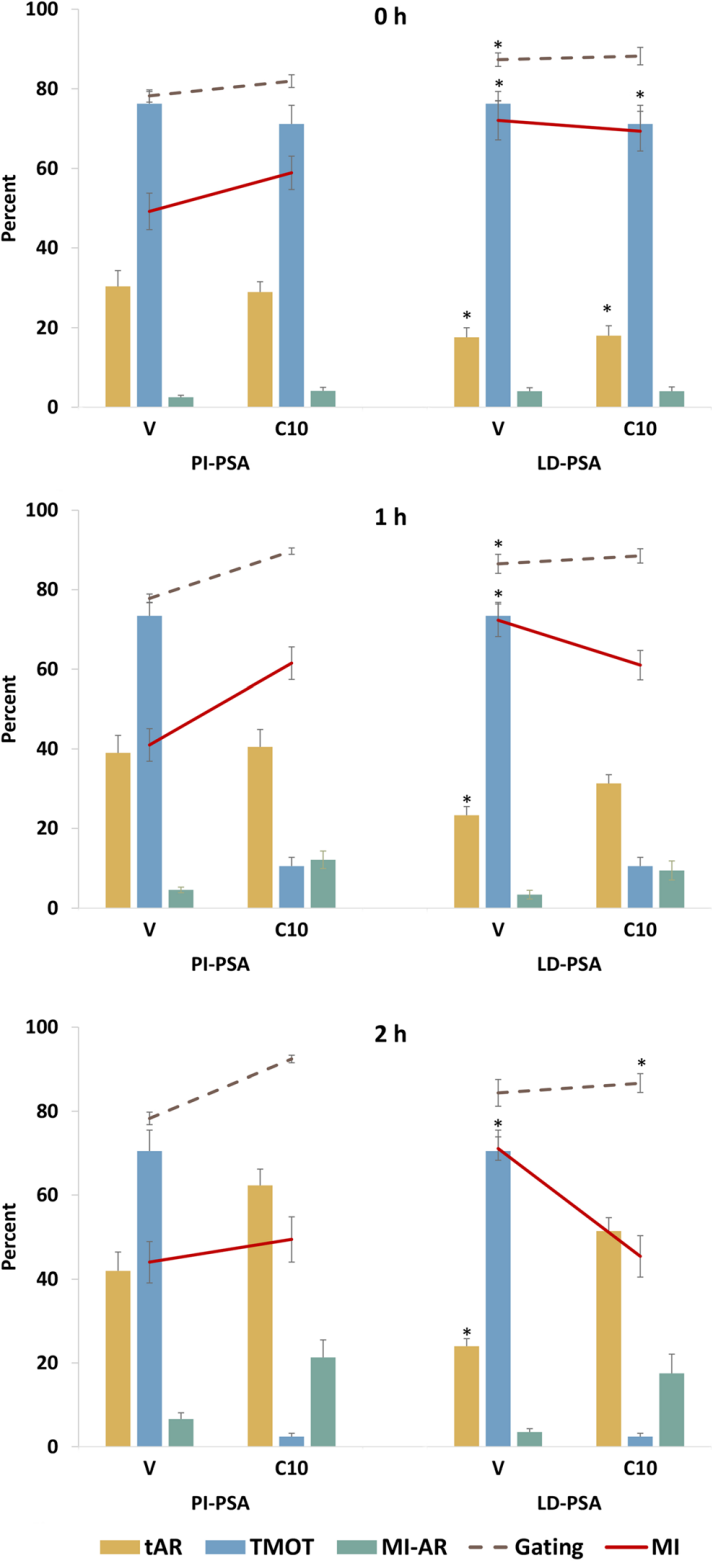
Comparison of results obtained with the PI-PSA vs. LD-PSA methods. The percentage of total sperm motility (TMOT) was measured by CASA and for clarity is shown in comparison to both PI-PSA and LD-PSA results. Membrane integrity (MI), total acrosome reaction (tAR), and membrane intact–acrosome reacted sperm (MI-AR) were assessed in samples exposed to 0 (V) or 10 μM A23187 (C10) for 10 min, followed by washing and incubation for 0, 1, or 2 h. Parameters for which the LD-PSA results differed significantly from the PI-PSA results (*P* < 0.05) are shown by asterisks. Note that membrane integrity agrees with total motility (i.e., MI is the same as or higher than TMOT) for LD-PSA but not for PI-PSA

**Fig. 6:**
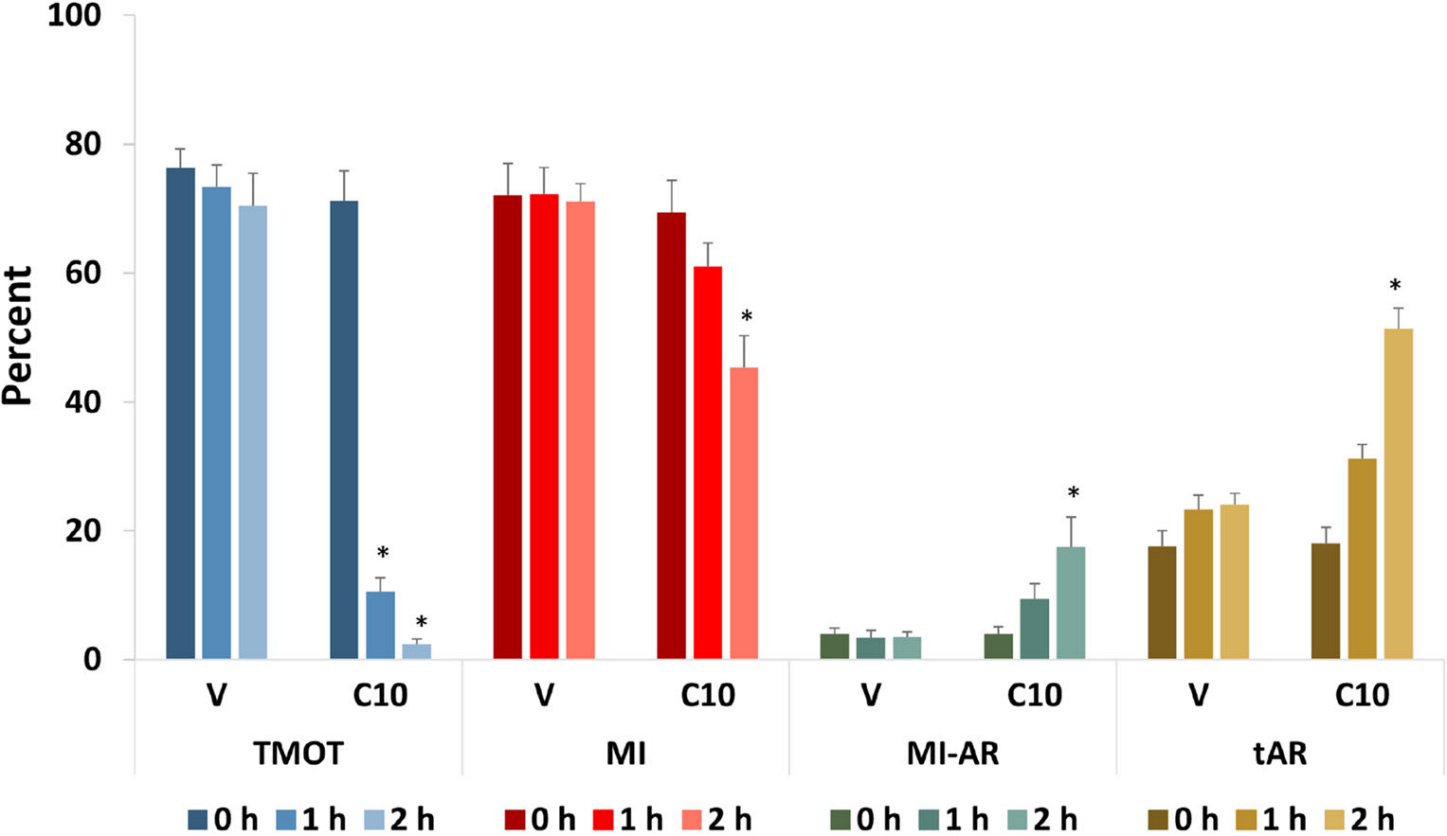
Effect of exposure of equine sperm to vehicle (V) or 10 μM A23187 (C10) for 10 min, followed by washing and incubation for 0, 1, or 2 h on total sperm motility (TMOT) as measured by CASA, and membrane integrity (MI), membrane intact-acrosome reacted sperm (MI-AR), and total acrosome reacted sperm (tAR) as assessed by the LD-PSA method. For each parameter, significant differences (*P* < 0.05) between vehicle and A23187-treated samples at the same timepoint are represented by asterisks. At 1 h, A23187 significantly decreased TMOT. At 2 h, A23187 treatment significantly decreased total motility and MI, and increased both MI-AR and tAR

Analysis via Bland-Altman plot (Suppl. Fig. 1) returned the same significance information as did ANOVA; therefore, only ANOVA results are presented. In this study, CASA evaluation of sperm motility showed that at 1 h and 2 h, TMOT was notably and significantly lower for A23187-treated than for vehicle-treated sperm (Figs. 5 and 6; discussed in more detail below). Figure 5 shows the comparison of measured sperm parameters (MI, tAR, and MI-AR) assessed by the two evaluated methods (PI-PSA and LD-PSA). On flow cytometry, recorded MI in V samples was significantly lower when sperm were assessed with the PI-PSA method than with the LD-PSA method at all timepoints. In V treatments, the MI measured by the PI-PSA method was lower than TMOT by 22.9, 31.3, and 27.1 percentage points at 0 h, 1 h, and 2 h, respectively. In contrast, the MI measured by the LD-PSA method was lower than TMOT by 4.2 and 1.1 percentage points at 0 h and 1 h, and higher than TMOT by 0.6 percentage points at 2 h. There was also a difference between PI-PSA and LD-PSA methods in the recorded tAR: Sperm assessed by PI-PSA showed significantly higher tAR values than did those assessed by LD-PSA at all time periods in the V treatment, and at 0 h in the C10 treatment. tAR increased over time in the V treatment as measured by PI-PSA (*P* < 0.05 for 2 h vs. 0 h) but did not increase over time in the V treatment as measured by the LD-PSA method. Gating was significantly lower for PI-PSA than for LD-PSA for V at 0 and 1 h, and for C10 at 0 h, indicating a lower proportion of individualized sperm in the PI-PSA method. There were no significant differences in the measured MI-AR between the two methods. The complete analysis is presented in Suppl. Table 3.

Based on the more appropriate relationship between TMOT and MI measured by the LD-PSA method, as detailed above, we selected the results generated by the LD-PSA method to examine the effect of A23187 on equine sperm membrane parameters over time (Fig. 6). Figure 6 also shows more clearly the dramatic reduction in TMOT over time induced by 10 min exposure to 10 μM A23187 (C10). At 2 h, TMOT was 2.4 ± 0.8% for C10, vs. 70.5 ± 5.0% for V. Treatment with A23187 also significantly lowered MI by 2 h. Despite this, the percentage of membrane-intact acrosome-reacted sperm (MI-AR) increased significantly at 2 h after A23187 exposure. The tAR also increased significantly in the C10 treatment at 2 h. There were no significant changes in any parameter (TMOT, MI, MI-AR, or tAR) in the V treatment over time.

### Experiment 2: Comparison of results with the LD-PSA anti-agglutination method under various protocol modifications, including storage for 2 days before assessment

**Fig. 7:**
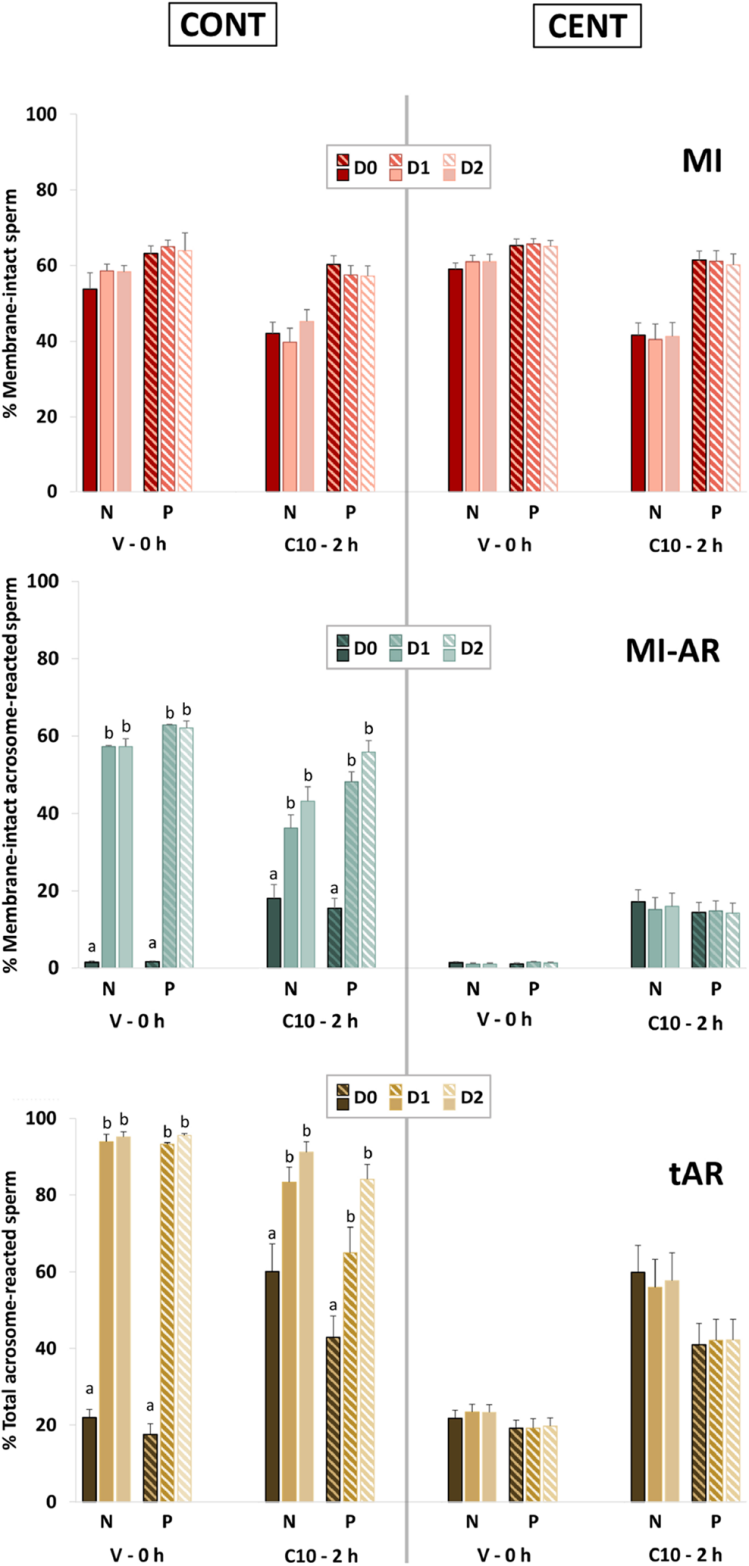
Effect of variations in the LD-PSA procedure on the percentages of sperm membrane integrity (MI), total acrosome reaction (tAR), and membrane intact acrosome reacted sperm (MI-AR). The procedural factors evaluated were absence (N) or presence (P) of 7 mg/mL bovine serum albumin (BSA) in the washing medium; performing the centrifugation to remove the stain and fixative after (CONT) or before (CENT) storage; and duration of storage (up to 6 h (D0), 24 h (D1), and 48 h (D2)). The values for the measured parameters MI, tAR, and MI-AR were compared among procedural variations under two conditions: Vehicle at 0 h and 10 μM A23187 at 2 h. Within treatments and conditions, significant differences (*P* < 0.05) between days are marked with an asterisk

Results for the control LD-PSA method (CONT) analyzed by flow cytometry on the same day the samples were fixed and stored (D0), i.e., the technique used in Exp. 1, were evaluated to assess the effect of 7 mg/mL BSA (P) during exposure to V or A23187 on measured parameters. Evaluation of these results (Fig. 7) showed that for the V-0 h treatment, there was no effect of P during processing and exposure on any parameter. For the C10-2 h treatment, samples processed and exposed to A23187 in P medium showed significantly higher MI (60.3 ± 2.3%) than did those processed and exposed in medium without BSA (42.0 ± 3.0% , *P* < 0.001; this significance is not indicated on the figure as the emphasis is on differences among days). Both t-AR and MI-AR were significantly higher in C10-2 h than in V-0 h samples for both media.

**Fig. 8:**
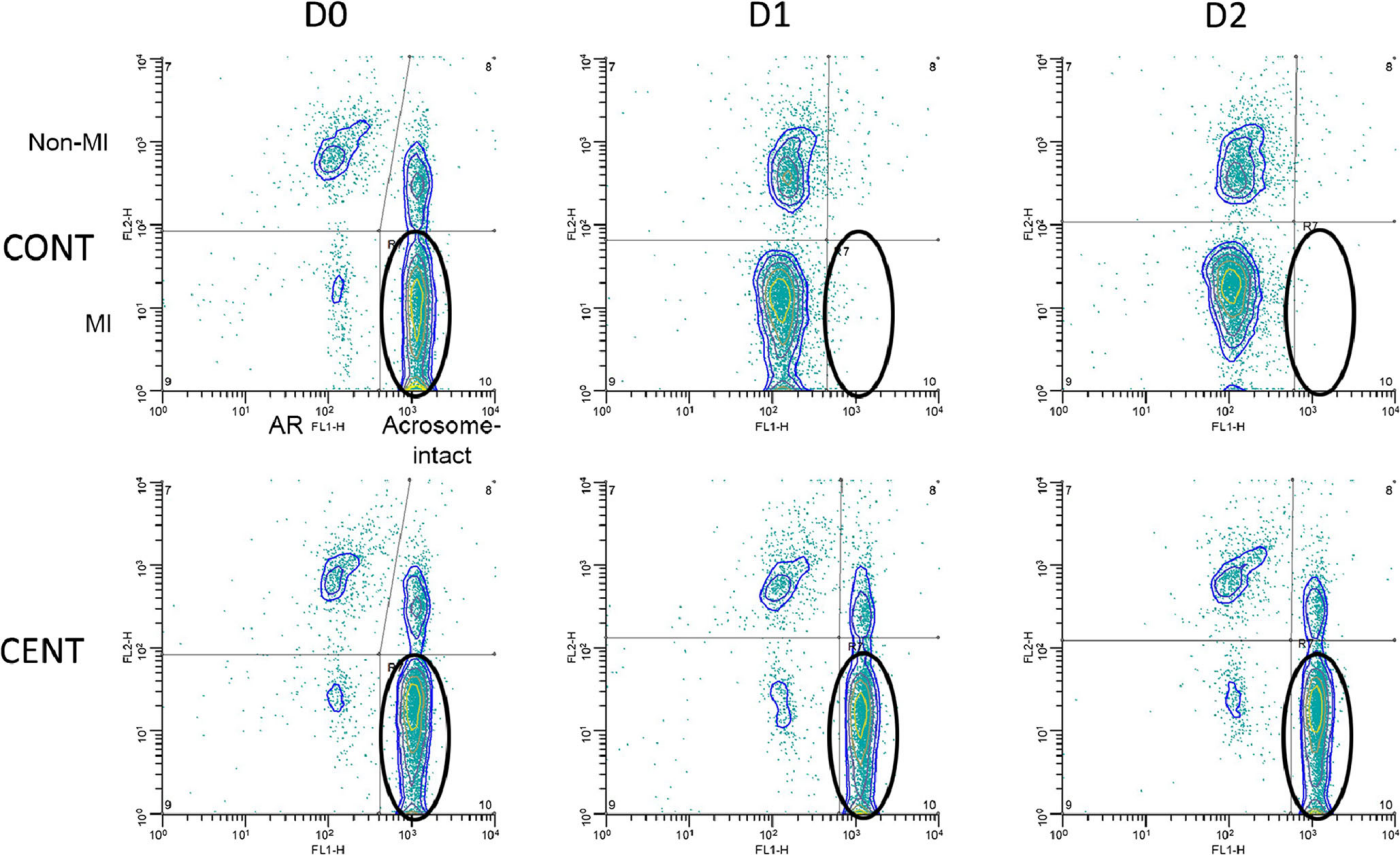
Representative scattergrams showing flow cytometry for membrane integrity and acrosome reaction of sperm from one stallion ejaculate processed using the LD-PSA method, over 0, 1, and 2 days of storage (0D, 1D, and 2D). The centrifugation to remove the stain and fixative was performed after (CONT) or before (CENT) storage. MI = membrane intact; AR = acrosome-reacted. There were no significant differences in MI, MI-AR, or total AR (tAR) between CONT and CENT at D0. For the CONT preparations, there was a significant decrease (*P* < 0.05) in membrane-intact, acrosome-intact sperm (oval) between D0 and the later days, and a concomitant increase in MI-AR and tAR. This indicated increased staining of the acrosome during storage in the presence of 2% paraformaldehyde and live/dead stain. There were no significant changes in any parameter over time for the CENT preparations

We then evaluated the effect of variations in the LD-PSA protocol on results. There were no differences in MI over the days of storage for either processing treatment (Fig. 7). Samples that were centrifuged before storage (CENT) were not different from samples processed by the control method (CONT) at D0. Samples processed using CENT showed no changes in any measured parameter over the 2 days of storage (D0–D2). In contrast, samples processed using CONT showed significantly and markedly higher MI-AR and tAR values at D1 and D2 than at D0. Bland-Altman plots for these comparisons are shown in Suppl. Fig. 2; the complete ANOVA results are presented in Suppl. Table 4. The changes in population distribution on flow cytometry scatter plots, for membrane integrity and acrosomal status over storage time in the CONT and CENT treatments for one C10-2 h replicate, are shown in Fig. 8.

## Discussion

In this report, using a novel method for membrane analysis (LD-PSA), we present new information on the physiology of equine sperm exposed to the calcium ionophore A23187. While the results outlining the response of equine sperm to A23187 are interesting, more important are our findings on the problems inherent with analysis of incubated sperm by flow cytometry.

The results of our studies show conclusively that the current standard method of analysis of sperm membrane integrity via flow cytometry, i.e., direct staining with propidium iodide and PSA (PI-PSA), can provide non-representative results for membrane integrity. In our first preliminary experiment, using the PI-PSA method, in the vehicle treatment the percentage of motile sperm was much higher than was the percentage of membrane-intact (viable) sperm. These results would indicate that a proportion of membrane-damaged (dead) sperm were motile, which should not be possible [23, 25]. We termed this phenomenon (i.e., that MI < TMOT) the “zombie sperm” dilemma.

When addressing the cause for the zombie sperm dilemma, we noted that the sperm in our studies that were resuspended in a capacitation-type medium (LPB) showed a high amount of agglutination. On microscopic evaluation, we noted that these sperm aggregates were formed largely of viable sperm, a finding noted previously by others [14, 20–22]. We hypothesized that, due to their large size, these aggregates of viable sperm were not included in the gated population analyzed on flow cytometry, and thus the remaining sperm that were analyzed (the individualized sperm) were not representative of the entire sperm population in that they were biased toward dead (non-membrane-intact) sperm.

In addition, we noted that when the sperm became immotile, they disaggregated. An advantage of the current experimental design for exploration of this phenomenon was that we were using a treatment (A23187) that induces immobility due to accumulation of excess intracellular calcium; however, these immobilized sperm initially remain viable [27, 28]. Thus, in our studies, there were two reasons that sperm became immotile: (1) because they were immobilized by A23187 treatment, but were still membrane-intact (viable); or (2) because they became membrane-damaged over time (dead).

These two hypotheses, i.e., that gating-out of sperm aggregates biases the reported measurements, and that sperm disaggregate when they lose motility, explain the seemingly illogical finding for MI as measured by PI-PSA that at time 0, MI increased with increasing A23187 dose. Following these hypotheses, it is likely that in sperm treated with high A23187 concentrations, the sperm became immotile, but they were still initially membrane-intact. These sperm disaggregated and became individualized; thus, these immotile but still viable sperm were able to be analyzed by flow cytometry. The measured MI appeared to be higher than in control sperm because the individualized population included membrane-intact, immotile sperm; whereas in controls, a proportion of viable sperm were aggregated and thus absent from the individualized population. Over more time, A23187-treated sperm underwent membrane damage (died) due to prolonged excess intracellular calcium. These sperm were individualized and thus included in the Gating, but they were not membrane-intact; thus, the measured MI decreased over time.

When initially exploring the zombie sperm dilemma (i.e., MI < TMOT), it was not clear whether the incongruous finding was a result of artificially high TMOT or of artificially low MI in samples with high agglutination. Performance of CASA also relies on analysis of individual sperm, and agglutination is generally accepted as affecting CASA results [14], although we could not find reports presenting data on this effect. Aggregates were present in samples assessed by CASA, as visualized on the associated sperm-tracking videos, and, as noted above, these aggregates appeared to be composed largely of motile sperm (sperm with vigorous tail movement). The CASA sperm-tracking function showed that some sperm within the aggregates, especially those aggregates in which sperm were less compact, were included in the motility analysis. Thus, two possibilities exist regarding a potential effect of agglutination in confounding CASA results: (A) that highly motile sperm aggregate; when in these aggregates they are less likely to be counted on CASA, resulting in artifactually low reported motility; or (B) that immotile sperm included in a moving aggregate might be counted as motile on CASA, resulting in artifactually high reported motility. In reviewing our data to clarify this point, we noted in Preliminary study B that in the vehicle treatment at time 0, the reported TMOT was slightly but non-significantly higher in LPB (an agglutinating medium) than in IN (a non-agglutinating medium; Fig. 3), which runs counter to hypothesis A above. These findings could support hypothesis B, or could indicate actual higher motility in LPB medium. In agreement with the possibility that LPB supports higher motility than does IN was the finding that VCL was significantly higher in LPB than in IN at 0 h (Suppl. Table 2). We developed the LPB medium, a novel medium, based on findings that HBSS supported greater longevity of motility in incubated equine sperm than did other tested basal media [3, 29]; we added BSA at a concentration (7 mg/mL) similar to that used in studies reporting successful equine IVF (3 to 10 mg/mL) and added lactate and pyruvate as metabolic substrates, as it has been shown that equine sperm depend largely on oxidative phosphorylation for generation of ATP [30, 31].

The finding that ascertained that the zombie sperm dilemma (MI < TMOT) was related to a low MI reading, rather than a high TMOT reading, was that at time 0, the measured MI of vehicle-treated sperm resuspended in LPB (49%) was absolutely and significantly lower than was the measured MI of the same sperm resuspended in IN (76%; Fig. 3). The data that established that this low MI was related to agglutination was that the finding of MI < TMOT was observed in the medium that promoted agglutination (LPB) but not in the medium that does not promote agglutination (IN, a milk-based semen extender especially designed for use in the stallion). In LPB, as sperm became immobilized by the action of A23187, they disaggregated (as shown by an increase in Gating) and the MI increased accordingly (Fig. 3). Sperm do not agglutinate in IN; thus in IN, while A23187 treatment induced a drastic reduction in motility, MI did not change; in IN, the MI was not significantly different between vehicle- and A23187-treated sperm at time 0 (MI > TMOT in both treatments). Over time (at 1 h and 2 h), the A23187-treated sperm in IN lost viability, possibly due to a more extreme calcium influx due to the higher calcium concentration in this extender, and measured MI then decreased (Fig. 3).

Previous studies have indicated that incubation in capacitation conditions (BSA, calcium, and bicarbonate) is associated with sperm agglutination [12, 13, 15–18]. In boar sperm, an anti-agglutinin produced in the epididymis is found on ejaculated sperm, and is released during incubation for capacitation, especially in the presence of serum [19]. Head-to-head agglutination in boar sperm is induced by bicarbonate and activation of cAMP, and requires calcium [32]. Agglutination appears to be related to changes in sperm membrane proteins; in ram sperm, agglutination is associated with formation of disulfide bonds on copper-binding sperm membrane proteins, hypothesized to be cell adhesion proteins of the disintegrin and metalloproteinase (ADAM) family [33]. With this understanding, we performed experiments to try to decrease this agglutination. First, we tried addition of the thiol D-penicillamine (PEN), which decreases agglutination of bull and ram sperm in capacitating-type medium, associated with reduction of disulfide bonds on membrane proteins [14, 20, 33], and has been used to protect stallion sperm from oxidative stress [34]. On analysis by CASA and PI-PSA, the addition of 1 mM PEN to the medium was not associated with a difference from control for any parameter at any timepoint. The lack of effect of PEN on agglutination in our study, in contrast to the findings of Leahy et al. in the ram [14], could possibly be due to the action of PEN as a chelator of metals, including copper [14, 34–36]. Ram sperm are highly sensitive to copper, and ovine serum, used as protein source by Leahy et al. [14], contains appreciable levels of copper [37].

The application of IN was a second approach to attempt to limit agglutination. This was done only to study the effect of agglutination, as use of IN does not allow individual evaluation of medium components associated with capacitation, one of our overall goals. As noted above, the amount of agglutination accordingly decreased, as reflected in a significantly higher gated population (Fig. 3) and concomitantly, the recorded % of MI sperm increased vis-à-vis the LPB control. The immediate precipitous drop in motility in sperm resuspended in IN after exposure to A23187 was unexpected. As noted above, this decrease in motility, and subsequent loss of viability, could be associated with the higher calcium concentration in this extender, especially as equine sperm appear to be relatively sensitive to increased calcium concentrations [25].

After finding PEN and milk-based extender either ineffective or problematic for use in our studies, we tried the cell-dissociating agent Accumax. As described by the vendor (Millipore Sigma), Accumax is a commercial solution of proteolytic and collagenolytic enzymes and DNAses, and other components, designed to prevent cell agglutination. We found that use of Accumax on fresh sperm resulted in sperm death (data not shown) likely due to digestion of sperm membranes by this solution. Therefore, we decided to utilize a fixable Live/Dead stain, to allow fixation of sperm after they had been stained to establish membrane status. Anti-agglutination measures could then be applied on the fixed sperm without affecting sperm membrane analysis. The Live/Dead stain uses dyes that fluoresce when covalently bound to amines. In membrane-damaged sperm, the dyes have access to amines in the cell interior, resulting in intense fluorescence. The reaction of the dye with amines is irreversible; thus, the discrimination is preserved after fixation [38, 39]. Because the sperm were fixed, we were then able to utilize Accumax as an anti-agglutination measure. In addition, a high concentration of Triton-X was added after fixation. Triton-X, a detergent, is utilized to complete the permeabilization of the acrosomal membrane begun by the paraformaldehyde, so that the acrosome, if present, can be completely visualized. Triton-X also has anti-agglutination activity, as it may decrease head-to-head membrane binding; however, it cannot be used on fresh sperm as it causes dissolution of equine sperm membranes at low concentrations [25]. In the LD-PSA protocol, we increased the Triton-X concentration to 10 times that needed for permeabilization, to increase the anti-agglutination effect. Fixable Live/Dead staining in combination with an acrosomal-binding lectin has been used on equine and bovine sperm [40, 41], but to the best of our knowledge, has not previously been incorporated with anti-agglutination measures to address the problem of agglutination. We found that use of the Live/Dead stain followed by fixation, treatment with Accumax and high-dose Triton-X, staining with PSA, and re-suspension in Accumax (the LD-PSA method) yielded data having an appropriate correspondence of MI with TMOT (Fig. 5).

Interestingly, PI-PSA and LD-PSA protocols showed significant differences not only in measured MI, as discussed for the zombie sperm dilemma, but also in total AR (tAR). This measure in vehicle-treated sperm was higher in PI-PSA than in LD-PSA at time 0 and 1 h (Fig. 5). This is logical, as in PI-PSA, due to agglutination of viable sperm, the individualized (analyzed) population is biased toward membrane-damaged sperm, which are more likely to be acrosome-reacted. In contrast to tAR, there were no significant differences for MI-AR between PI-PSA and LD-PSA methods. This is likely associated with the fact that in our study, appreciable proportions of MI-AR sperm were seen only after A23187 treatment, which also caused sperm to be immobilized. The immotile sperm disaggregated and became individualized, thus eliminating the effect of agglutination, and so the population included in the Gating (the population included in the analysis) was similar for both PI-PSA and LD-PSA assessment methods.

As the LD-PSA method appeared to provide representative results, and might be adopted for general use, we explored the effect of procedural variations for this novel assessment method on measured sperm membrane integrity and acrosome status. There was no effect of sperm storage on MI, supporting previous findings that Live/Dead-stained equine sperm samples could be stored after fixation for up to 5 days, with only minor (about 10% loss in MI/day) effects on viability results [42]. We based our initial protocol of Live/Dead staining on this previous report [42]; because those authors wanted to utilize the stain on sperm collected by referring veterinarians in the field, the fixed samples were stored without centrifuging to remove the dye and paraformaldehyde, to assess the effects of storage. While, as per this previous report, using this methodology we did not find an effect of storage on MI, we found in Exp. 2 that storage in the presence of the dye and paraformaldehyde (i.e., without centrifugation and resuspension before storage) for 1 or 2 days (D1 and D2) resulted in apparent loss of the acrosome, as shown by a significant and marked increase in the proportion of sperm classified as acrosome-reacted. This change over time was prevented if the paraformaldehyde and dye was removed by centrifugation before storage, as recommended by the manufacturer of the Live/Dead stain; we recommend that this precaution be taken when the method is applied to sperm that will not be analyzed immediately. The ability to store sperm for later analysis is of profound value in allowing assessment of sperm sampled at multiple timepoints, or at satellite facilities.

The results of our analyses in these studies do provide new data on the effect of calcium ionophore treatment on equine sperm. To the best of our knowledge, this is the first study in which the effects of A23187 on motility, and on proportion of acrosome-reacted viable sperm, have been objectively investigated in equine sperm under the conditions in which A23187 has been used for IVF, i.e., brief exposure of sperm to the ionophore followed by washing and incubation in a capacitation-type medium [3–6]. With this protocol, we were able to repeatedly generate a population of up to 20% membrane-intact, acrosome reacted sperm. We found that equine sperm did not respond to exposure for 10 min to 1 μM A23187 (Fig. 1) but did respond to concentrations of 5 and 10 μM A23187, showing immediate significant loss of motility. Motility decreased over time to almost zero after 2 h incubation. Total motility decreased more rapidly when sperm were exposed to A23187 in medium without BSA; this is expected as albumin binds A23187 and reduces A23187-induced calcium influx in equine sperm [43]. There was an increase in the population of membrane-intact, acrosome-reacted sperm, but only at 2 h after ionophore exposure. Indeed, through all the studies, it appeared that the motility decreased in direct relation to the ability of that treatment to induce the acrosome reaction (Figs. 1 and 6). This suggests that one of the factors that may be related to the low rate of equine IVF associated with sperm treatment with A23187 could be that for treatments capable of stimulating the acrosome reaction, sperm lose motility before the acrosome reactions initiated by the treatment occur.

Notably, sperm that were not treated with ionophore (vehicle treatment) but were incubated in capacitation-type media (LPB: presence of BSA, calcium and bicarbonate) for 2 h showed no changes in any parameter (TMOT, PMOT, MI, MI-AR, or tAR) over time. This reflects the inability to induce capacitation-like changes in equine sperm under conditions that would stimulate this change in other species. This lack of capacitating effect has been associated with the inefficiency of equine IVF [2, 44].

In summary, the finding that sperm agglutination significantly affects the results of flow cytometric evaluation is important to consider when performing sperm evaluations. This is especially relevant to sperm incubated under capacitating conditions, as these conditions are associated with increased sperm agglutination. When no measures were taken to reduce agglutination (PI-PSA method), the measured proportion of membrane-intact sperm was markedly lower than was total motility as measured by CASA, indicating that the results were not representative of the population being assessed. Our conclusions after performing these studies are that viable, motile equine sperm incubated in capacitating conditions tend to aggregate, and that as sperm lose motility due to either membrane damage or, in our studies, A23187-induced calcium overload, they disaggregate. These two factors differentially affect measurements on flow cytometry, in which only individualized sperm are assessed. Methods to reduce sperm agglutination before flow cytometry was performed (LD-PSA method) resulted in more correspondent MI data. These findings are extremely important in relation to studies assessing sperm membrane integrity.

## Supplementary Information

ESM 1 (DOCX 35 kb)

ESM 2 (PDF 329 kb)
