# Supplementary material for: Flow-cytometric analysis of membrane integrity of stallion sperm in the face of agglutination: the “zombie sperm” dilemma
Source: J Assist Reprod Genet. 2021 May 15;38(9):2465–80. doi: 10.1007/s10815-021-02134-z (PMC8490572; doi:10.1007/s10815-021-02134-z)
Supplement: Supplementary file 1 — (DOCX 35 kb) [file 10815_2021_2134_MOESM1_ESM.docx]

**Supplementary Table 1. Motility (assessed by CASA) and plasma membrane integrity and acrosome status in equine sperm treated with A23187 as assessed by the standard flow cytometry method (PI-PSA) under various conditions (0 vs 7mg/mL BSA and 0-10 µM A23187).**

| **0 h** | **TMOT** | **PMOT** | **MI** | **MI-AR** | **tAR** |
| --- | --- | --- | --- | --- | --- |
| **N-V** | 73.7 ± 7.6^a^ | 28.8 ± 8.9^a^ | 49.4 ± 7.4 ^ab^ | 3.4 ± 1.3^a^ | 34.2 ± 5.6 ^a^ |
| **N-C1** | 72.8 ± 7.9^a^ | 32.0 ± 8.9 ^a^ | 57.8 ± 6.4 ^ab^ | 3.6 ± 1.0^a^ | 32.4 ± 6.9 ^a^ |
| **N-C5** | 53.55 ± 7.0^ab^ | 13.6 ± 2.6^b^ | 66.8 ± 6.6 ^a^ | 4.3 ± 1.3^a^ | 28.8 ± 5.9 ^a^ |
| **N-C10** | 32.2 ± 12.2^b^ | 3.0 ± 0.9^cd^ | 70.4 ± 3.9 ^a^ | 4.6 ± 1.5^a^ | 25.2 ± 4.0 ^a^ |
| **P-V** | 75.5 ± 7.4^a^ | 33.8 ± 8.1 ^a^ | 46.0 ± 7.5 ^ab^ | 4.5 ± 2.1^a^ | 35.5 ± 8.8 ^a^ |
| **P-C1** | 79.8 ± 6.0^a^ | 32.5 ± 8.4 ^a^ | 47.3 ± 8.1 ^ab^ | 5.4 ± 2.3 ^a^ | 39.0 ± 9.1 ^a^ |
| **P-C5** | 76.8 ± 7.6^a^ | 35.2 ± 7.7 ^a^ | 46.8 ±8.3 ^ab^ | 5.6 ± 2.4 ^a^ | 39.8 ± 9.3 ^a^ |
| **P-C10** | 72.3 ± 10.5^a^ | 33.2 ± 7.6 ^a^ | 53.9 ± 8.8 ^a^ | 6.4 ± 2.1 ^a^ | 36.9 ± 6.7 ^a^ |
|  |  |  |  |  |  |
| **0.5 h** | **TMOT** | **PMOT** | **MI** | **MI-AR** | **tAR** |
| **N-V** | 69.5 ± 6.2^a^ | 28.7 ± 5.2 ^a^ | 43.6 ± 7.1 ^ab^ | 3.6 ± 0.8 ^a^ | 43.1 ± 9.6 ^a^ |
| **N-C1** | 69.5 ± 8.8^a^ | 28.8 ± 7.2 ^a^ | 41.4 ± 9.7 ^ab^ | 3.3 ± 1.2 ^a^ | 42.8 ± 10.0 ^a^ |
| **N-C5** | 35.4 ± 13.6^b^ | 2.2 ± 0.3^cd^ | 56.0 ± 7.2 ^a^ | 4.6 ± 1.5 ^a^ | 33.4 ± 8.1 ^a^ |
| **N-C10** | 28.2 ± 13.1^b^ | 0.7 ± 0.5^d^ | 51.9 ± 5.7 ^a^ | 7.8 ± 3.3 ^a^ | 40.9 ± 9.1 ^a^ |
| **P-V** | 71.7 ± 9.2^a^ | 31.7 ± 5.5 ^a^ | 38.4 ± 6.8 ^ab^ | 4.9 ± 1.5 ^a^ | 43.1 ± 6.7 ^a^ |
| **P-C1** | 71.6 ± 7.0^a^ | 32.2 ± 7.7 ^a^ | 39.9 ± 5.6 ^ab^ | 5.6 ± 2.4 ^a^ | 45.6 ± 6.6 ^a^ |
| **P-C5** | 61.1± 9.1^ab^ | 26.0 ± 4.9 ^a^ | 51.1 ± 8.9 ^ab^ | 8.8 ± 3.5 ^a^ | 44.0 ± 5.6 ^a^ |
| **P-C10** | 29.7 ± 7.4^b^ | 5.7 ± 1.8^c^ | 62.7 ± 7.7 ^a^ | 8.8 ± 2.5 ^a^ | 36.6 ± 4.5 ^a^ |
|  |  |  |  |  |  |
| **1 h** | **TMOT** | **PMOT** | **MI** | **MI-AR** | **tAR** |
| **N-V** | 24.1 ± 12.1^b^ | 28.2 ± 5.9 ^a^ | 36.9 ± 5.9^ab^ | 3.6 ± 1.0 ^a^ | 44.2 ± 8.2 ^a^ |
| **N-C1** | 14.3 ± 7.7^bc^ | 25.5 ± 6.6 ^a^ | 40.8 ± 6.4^ab^ | 5.5 ± 1.9 ^a^ | 46.1 ± 8.9 ^a^ |
| **N-C5** | 27.7 ± 13.9^b^ | 1.3 ± 0.3^cd^ | 48.3 ± 7.5^ab^ | 8.7 ± 4.0 ^a^ | 46.8 ± 11.3 ^a^ |
| **N-C10** | 18.0 ± 9.0^bc^ | 0.5 ± 0.3c^d^ | 34.9 ± 6.6^b^ | 9.2 ± 3.3 ^a^ | 59.9 ± 12.5^ab^ |
| **P-V** | 69.8 ± 8.7^a^ | 30.0 ± 5.2 ^a^ | 35.4 ± 7.6^b^ | 5.0 ± 1.6 ^a^ | 46.9 ± 7.9 ^a^ |
| **P-C1** | 69.8 ± 8.9^a^ | 31.7 ± 6.2 ^a^ | 34.7 ± 9.3^b^ | 6.0 ± 1.6 ^a^ | 52.6 ± 7.6 ^ab^ |
| **P-C5** | 43.8 ± 9.4^ab^ | 9.4 ± 4.8^c^ | 55.2 ± 4.6^ab^ | 11.0 ± 1.6^ab^ | 43.1 ± 4.5 ^a^ |
| **P-C10** | 13.3 ± 5.7^bc^ | 1.7 ± 1.0^cd^ | 51.2 ± 8.6^ab^ | 10.2 ± 0.8^ab^ | 46.2 ± 8.9 ^a^ |
|  |  |  |  |  |  |
| **2 h** | **TMOT** | **PMOT** | **MI** | **MI-AR** | **tAR** |
| **N-V** | 17.7 ± 9.9^bc^ | 12.2 ± 6.1^b^ | 35.2 ± 6.9^b^ | 5.4 ± 1.2 ^a^ | 50.2 ± 5.2 ^a^ |
| **N-C1** | 28.8 ± 10.8^b^ | 18.1 ± 9.0^ab^ | 39.9 ± 7.5^ab^ | 6.8 ± 2.2 ^a^ | 49.5 ± 7.3 ^a^ |
| **N-C5** | 11.0 ± 5.8^c^ | 0.3 ± 0.1^d^ | 27.8 ± 9.5^bc^ | 9.3 ± 2.6^ab^ | 70.7 ± 7.5^bc^ |
| **N-C10** | 10.5 ± 3.2^c^ | 0.4 ± 0.2^d^ | 18.0 ± 6.1^bc^ | 7.2 ± 1.6^a^ | 77.4 ± 5.4^c^ |
| **P-V** | 67.6 ± 9.9^a^ | 29.9 ± 5.7 ^a^ | 33.5 ± 9.1^b^ | 7.5 ± 1.6 ^a^ | 51.1 ± 7.3 ^ab^ |
| **P-C1** | 65.4 ± 10.8^a^ | 27.1 ± 8.6 ^a^ | 35.3 ± 9.2^b^ | 8.8 ± 2.3 ^a^ | 55.7 ± 6.1^ab^ |
| **P-C5** | 11.0 ± 5.8^c^ | 1.7 ± 0.6^cd^ | 41.6 ± 10.5^ab^ | 14.0 ± 2.0^b^ | 60.6 ± 10.5^b^ |
| **P-C10** | 8.8 ± 3.2^c^ | 0.6 ± 0.2^d^ | 39.9 ± 12.1^ab^ | 14.9 ± 2.5^b^ | 62.1 ± 10.0^b^ |

N= Sperm exposed to A23187 in 0 mg/ml BSA medium; P = Sperm exposed to A23187 in 7 mg/ml BSA medium; TMOT = Sperm total motility; PMOT = Sperm progressive motility; MI = membrane integrity; MI-AR= membrane intact-acrosome reacted sperm; tAR = total acrosome reaction. Values are expressed as mean ± SEM. Within columns (including all time periods combined), different superscripts indicate significantly different values (P < 0.05).

**Supplementary Table 2. Effect of anti-agglutinant measures (addition of D-penicillamine or use of milk-based extender) after A23187 exposure on sperm motility (measured by CASA) and on viability and acrosome status as assessed by the standard flow cytometry method (PI-PSA)**

| **0 h** | **Gating** | **TMOT** | **PMOT** | **MI** | **MI-AR** | **tAR** |
| --- | --- | --- | --- | --- | --- | --- |
| **N-V** | 78.2 ± 1.5^b^ | 76.3 ± 3.0^a^ | 27.5 ± 3.1^b^ | 49.2 ± 4.6^bc^ | 2.5 ± 0.5^a^ | 30.3 ± 4.0^b^ |
| **N-C10** | 81.9 ± 1.6^b^ | 71.2 ± 4.7^a^ | 27.5 ± 3.4^b^ | 58.9 ± 4.2^b^ | 4.1 ± 0.9^a^ | 28.9 ± 2.6^b^ |
| **N - V - IN** | 91.4 ± 1.0^a^ | 58.1 ± 4.3^b^ | 50.9 ± 5.3^a^ | 76.0 ± 2.4^a^ | 1.9 ± 0.2^a^ | 16.0 ± 2.2^c^ |
| **N - C10 - IN** | 91.9 ± 0.9^a^ | 14.8 ± 7.2^c^ | 6.8 ± 4.3^c^ | 75.4 ± 2.7^a^ | 2.7 ± 0.2^a^ | 16.2 ± 2.1^c^ |
| **N - V - PEN** | 76.4 ± 1.2^b^ | 69.1 ± 4.2^a^ | 19.1 ± 4.1^b^ | 51.2 ± 5.1^bc^ | 2.7 ± 0.6^a^ | 27.6 ± 3.8^b^ |
| **N - C10 - PEN** | 79.2 ± 1.7^b^ | 60.9 ± 4.8^a^ | 20.6 ± 5.3^b^ | 57.4 ± 4.2^bc^ | 3.4 ± 1.1^a^ | 27.9 ± 3.1^b^ |
|  |  |  |  |  |  |  |
| **1 h** | **Gating** | **TMOT** | **PMOT** | **MI** | **MI-AR** | **tAR** |
| **N-V** | 77.8 ± 1.1^b^ | 73.4 ± 3.4^a^ | 29.6 ± 2.6^b^ | 41.0 ± 4.1^c^ | 4.6 ± 0.7^a^ | 39.0 ± 4.4^b^ |
| **N-C10** | 89.7 ± 0.8^b^ | 10.5 ± 2.2^c^ | 1.2 ± 0.4^d^ | 61.5 ± 4.1^b^ | 12.1 ± 2.2^ab^ | 40.5 ± 4.4^b^ |
| **N - V - IN** | 91.5 ± 0.8^a^ | 50.9 ± 5.3^b^ | 26.8 ± 6.1^b^ | 72.8 ± 2.5^a^ | 1.8 ± 0.3^a^ | 18.2 ± 2.2^c^ |
| **N - C10 - IN** | 93.4 ± 0.7^a^ | 0.4 ± 0.2^d^ | 0.1 ± 0.0^d^ | 42.0 ± 2.8^c^ | 13.9 ± 1.5^ab^ | 42.1 ± 2.8^b^ |
| **N - V - PEN** | 77.9 ± 1.4^b^ | 67.9 ± 4.0^a^ | 21.2 ± 3.2^b^ | 41.8 ± 3.7^c^ | 3.4 ± 1.4^a^ | 39.1 ± 4.2^b^ |
| **n - C10 - PEN** | 87.4 ± 2.1^ab^ | 15.4 ± 2.7^c^ | 1.8 ± 0.6^d^ | 57.5 ± 4.9^bc^ | 10.5 ± 2.9^ab^ | 37.4 ± 3.9^b^ |
|  |  | | | | |  |
| **2 h** | **Gating** | **TMOT** | **PMOT** | **MI** | **MI-AR** | **tAR** |
| **N-V** | 78.3 ± 1.5^b^ | 70.5 ± 5.0^a^ | 23.7 ± 2.9^b^ | 44.0 ± 4.9^c^ | 6.6 ± 1.5^a^ | 41.9 ± 4.5 |
| **N-C10** | 92.4 ± 0.9^a^ | 2.4 ± 0.8^d^ | 0.4 ± 0.1^d^ | 49.4 ± 5.4^bc^ | 21.3 ± 4.2^a^ | 62.3 ± 3.9^a^ |
| **N - V - IN** | 91.7 ± 0.8^a^ | 46.4 ± 6.1^b^ | 22.1 ± 5.3^b^ | 71.3 ± 2.5^a^ | 1.7 ± 0.2^a^ | 17.6 ± 2.0^c^ |
| **N - C10 - IN** | 93.8 ± 0.7^a^ | 0.5 ± 0.2^d^ | 0.2 ± 0.1^d^ | 32.3 ± 3.0^c^ | 17.6 ± 1.0^a^ | 54.0 ± 2.9^a^ |
| **N - V - PEN** | 81.3 ± 2.7^b^ | 69.9 ± 4.8^a^ | 22.7 ± 3.0^b^ | 44.9 ± 3.7^bc^ | 5.2 ± 1.7^a^ | 42.3 ± 4.5^b^ |
| **N - C10 - PEN** | 89.4 ± 1.2^ab^ | 2.7 ± 0.9^d^ | 0.3 ± 0.1^d^ | 47.6 ± 4.1^bc^ | 17.2 ± 5.4^a^ | 59.7 ± 4.1^a^ |

N= Sperm exposed to A23187 in 0 mg/ml BSA medium. Gating =(% of gated events) refers to the proportion of the total events that, based on forward scatter/side scatter, were considered to represent individual sperm, and thus were included in the analysis TMOT = Sperm total motility; PMOT = Sperm progressive motility; MI = membrane integrity; MI-AR= membrane intact-acrosome reacted sperm; tAR = total acrosome reaction. Values are expressed as mean ± SEM. Within columns (including all time periods combined), different superscripts indicate significantly different values (P < 0.05)

**)**

**Supplementary Table 3. Comparison of measured sperm parameters as assessed by PI-PSA vs. LD-PSA methods in sperm treated with A23187 and incubated for 0, 1 or 2 h**

| **0 h** | | **TMOT** | **Gating** | **MI** | **MI-AR** | **tAR** |
| --- | --- | --- | --- | --- | --- | --- |
| **V** | **PI - PSA** | 76.3 ± 3.0^a^ | 78.2 ± 1.5^b^ | 49.2 ± 4.6^b^ | 2.5 ± 0.5^c^ | 30.3 ± 4.0^bc^ |
|  | **LD -PSA** |  | 87.3 ± 1.7^a^ | 72.1 ± 4.9^a^ | 4.0 ± 0.9^c^ | 17.6 ± 2.4^c^ |
| **C10** | **PI - PSA** | 71.2 ± 4.7^a^ | 81.9 ± 1.6^a^ | 58.9 ± 4.2^ab^ | 4.1 ± 0.9^c^ | 28.9 ± 2.6^bc^ |
|  | **LD -PSA** |  | 88.2 ± 2.2^a^ | 69.4 ± 5.0^a^ | 4.0 ± 1.1^c^ | 18.0 ± 2.5^c^ |
| **1 h** | | **TMOT** | **Gating** | **MI** | **MI-AR** | **tAR** |
| **V** | **PI - PSA** | 73.4 ± 3.4^a^ | 77.8 ± 1.1^b^ | 41.0 ± 4.1^b^ | 4.6 ± 0.7^c^ | 39.0 ± 4.4^b^ |
|  | **LD -PSA** |  | 86.5 ± 2.4^a^ | 72.3 ± 4.1^a^ | 3.4 ± 1.1^c^ | 23.3 ± 2.2^bc^ |
| **C10** | **PI - PSA** | 10.5 ± 2.2^b^ | 89.7 ± 0.8^a^ | 61.5 ± 4.1^ab^ | 12.1 ± 2.2^b^ | 40.5 ± 4.4 ^b^ |
|  | **LD -PSA** |  | 88.5 ± 1.8^a^ | 61.0 ± 3.7^ab^ | 9.4 ± 2.4^b^ | 31.3 ± 2.2^bc^ |
| **2 h** | | **TMOT** | **Gating** | **MI** | **MI-AR** | **tAR** |
| **V** | **PI - PSA** | 70.5 ± 5.0^a^ | 78.3 ± 1.5^b^ | 44.0 ± 4.9^b^ | 6.6 ± 1.5^bc^ | 41.9 ± 4.5^b^ |
|  | **LD -PSA** |  | 84.4 ± 3.2^a^ | 71.1 ± 2.8^a^ | 3.5 ± 0.8^c^ | 24.0 ± 1.8^bc^ |
| **C10** | **PI - PSA** | 2.4 ± 0.8^c^ | 92.4 ± 0.9^a^ | 49.4 ± 5.4^b^ | 21.3 ± 4.2^a^ | 62.3 ± 3.9^a^ |
|  | **LD -PSA** |  | 86.7 ± 2.2^a^ | 45.4 ± 4.9^b^ | 17.5 ± 4.6^ab^ | 51.4 ± 3.2^a^ |

TMOT = Sperm total motility; Gating =(% of gated events) refers to the proportion of the total events that, based on forward scatter/side scatter, were considered to represent individual sperm, and thus were included in the analysis; MI = membrane integrity; MI-AR= membrane intact-acrosome reacted sperm; tAR = total acrosome reaction. Values are expressed as mean ± SEM. Within columns (including all time periods combined), different superscripts indicate significantly different values (P < 0.05)

**Supplementary Table 4.** **Comparison of results with the LD-PSA anti-agglutination method under various protocol modifications, including storage for up to two days before assessment**.

|  | **D0** | | | **D1** | | | **D2** | | |
| --- | --- | --- | --- | --- | --- | --- | --- | --- | --- |
|  | **MI** | **MI-AR** | **tAR** | **MI** | **MI-AR** | **tAR** | **MI** | **MI-AR** | **tAR** |
| **N - V - 0 h -CONT** | 53.7 ± 4.4^a^ | 1.5 ± 0.3^b^ | 22.0 ± 2.1^b^ | 58.5 ± 1.9^a^ | 57.3 ± 2.0^a^ | 93.9 ± 2.0^a^ | 58.3 ± 1.7^a^ | 57.3 ± 2.0^a^ | 95.2 ± 1.3^a^ |
| **N - V - 0 h - CENT** | 59.0 ± 1.7^a^ | 1.4 ± 0.2^b^ | 21.8 ± 2.1^b^ | 61.0 ± 1.7^a^ | 1.1 ± 0.2^c^ | 23.4 ± 2.0^c^ | 61.0 ± 2.0^a^ | 1.1 ± 0.2^c^ | 23.3 ± 2.0^c^ |
| **N - C10 - 2 h - CONT** | 42.0 ± 3.0^b^ | 18.1 ± 3.5^a^ | 60.1 ± 7.2^a^ | 39.8 ± 3.6^b^ | 36.2 ± 4.1^b^ | 83. 4 ± 3.9^a^ | 45.2 ± 3.1^b^ | 43.2 ± 3.7^a^ | 91.2 ± 2.7^a^ |
| **N - C10 - 2 h - CENT** | 41.5 ± 3.3^b^ | 17.2 ± 3.1^a^ | 59.9 ± 7.0^a^ | 40.5 ± 4.0^b^ | 15.2 ± 3.2^c^ | 56.0 ± 7.3^b^ | 41.2 ± 3.7^b^ | 16.0 ± 3.4^b^ | 57.7 ± 7.3^b^ |
| **P - V - 0 h - CONT** | 63.2 ± 2.0^a^ | 1.6 ± 0.2^b^ | 17.5 ± 2.8^b^ | 65.0 ± 1.7^a^ | 62.8 ± 2.0^a^ | 93.3 ± 1.4 ^a^ | 63.9 ± 4.7^a^ | 62.1 ± 1.8^a^ | 95.6 ± 0.5^a^ |
| **P - V - 0 h - CENT** | 65.3 ± 1.7^a^ | 1.1 ± 0.2^b^ | 19.2 ± 2.1^b^ | 65.7 ± 1.4^a^ | 1.5 ± 0.2^d^ | 19.2 ± 2.5^c^ | 65.1 ± 1.5^a^ | 1.3 ± 0.2^c^ | 19.7 ± 2.2^c^ |
| **P - C10 - 2 h - CONT** | 60.3 ± 2.3^a^ | 15.5 ± 2.6^a^ | 42.9 ± 5.6^a^ | 57.5 ± 2.5^a^ | 48.1 ± 5.9^ab^ | 84.1 ± 6.6 ^a^ | 57.2 ± 2.7^a^ | 55.8 ± 3.0^a^ | 95.0 ± 3.9^a^ |
| **P - C10 - 2 h - CENT** | 61.4 ± 2.4^a^ | 14.4 ± 2.6^a^ | 41.0 ± 5.6^a^ | 61.1 ± 2.8^a^ | 14.8 ± 2.5^c^ | 42.1 ± 5.5^b^ | 60.2 ± 2.9^a^ | 14.2 ± 2.6^b^ | 42.2 ± 5.4^b^ |

N= Sperm exposed to A23187 in 0 mg/ml BSA medium; P = Sperm exposed to A23187 in 7 mg/ml BSA medium; V – 0h = Sperm exposed to 0 µM A23187 for 10 min, washed, resuspended in LPB medium and stained after 0 h of incubation; C10 – 2h = Sperm exposed t0 10 µM A23187 for10 min, washed, resuspended in LPB medium and stained after 2 h of incubation; CONT = two centrifugations after refrigerated storage; CENT = split centrifugation, i.e. the first post-staining centrifugation and resuspension in DPBS(--)B was performed before placement into refrigerated storage, and the second centrifugation after removal from storage; D0, D1, D2 = Samples were analyzed by flow cytometry 0, 1 or 2 days after cold storage; MI = membrane integrity; MI-AR= membrane intact-acrosome reacted sperm; tAR = total acrosome reaction. Values are expressed as mean ± SEM. Within columns (and within day of assessment after storage), different superscripts indicate significantly different values (P < 0.05).
