# Supplementary material for: Flow-cytometric analysis of membrane integrity of stallion sperm in the face of agglutination: the “zombie sperm” dilemma
Source: J Assist Reprod Genet. 2021 May 15;38(9):2465–80. doi: 10.1007/s10815-021-02134-z (PMC8490572; doi:10.1007/s10815-021-02134-z)
Supplement: Supplementary file 2 — (PDF 329 kb) [file 10815_2021_2134_MOESM2_ESM.pdf]

Supplementary Figure 1

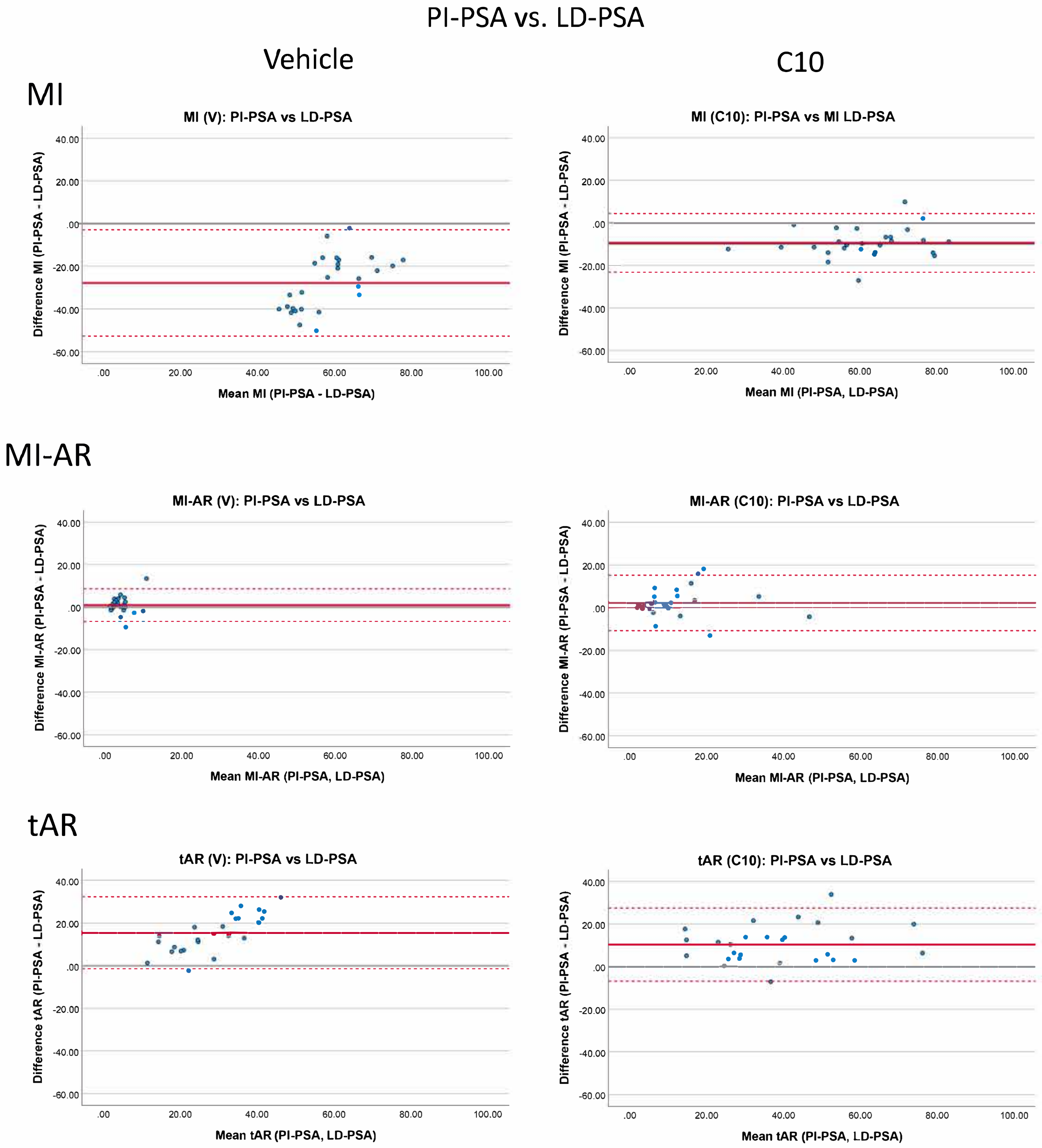

Supplementary Figure 1: Bland-Altman plots showing the agreement in the analysis of the percentage of membrane intact (MI), membrane-intact acrosome-reacted (MI-AR) and total acrosome-reacted (tAR) sperm between the assessment methods PI-PSA and LD-PSA in samples exposed to vehicle or 10 μM A23187 for 10 minutes, then washed and incubated for 0, 1 or 2 hours. Each of the paired measures is represented by assigning the mean of the two values on the X axis and the difference between the two values on the Y axis. The solid red line represents the mean difference (bias), and the two dashed red lines the 95% limits of agreement (± 1.96 standard deviations from the mean difference).

Supplementary Figure 2A

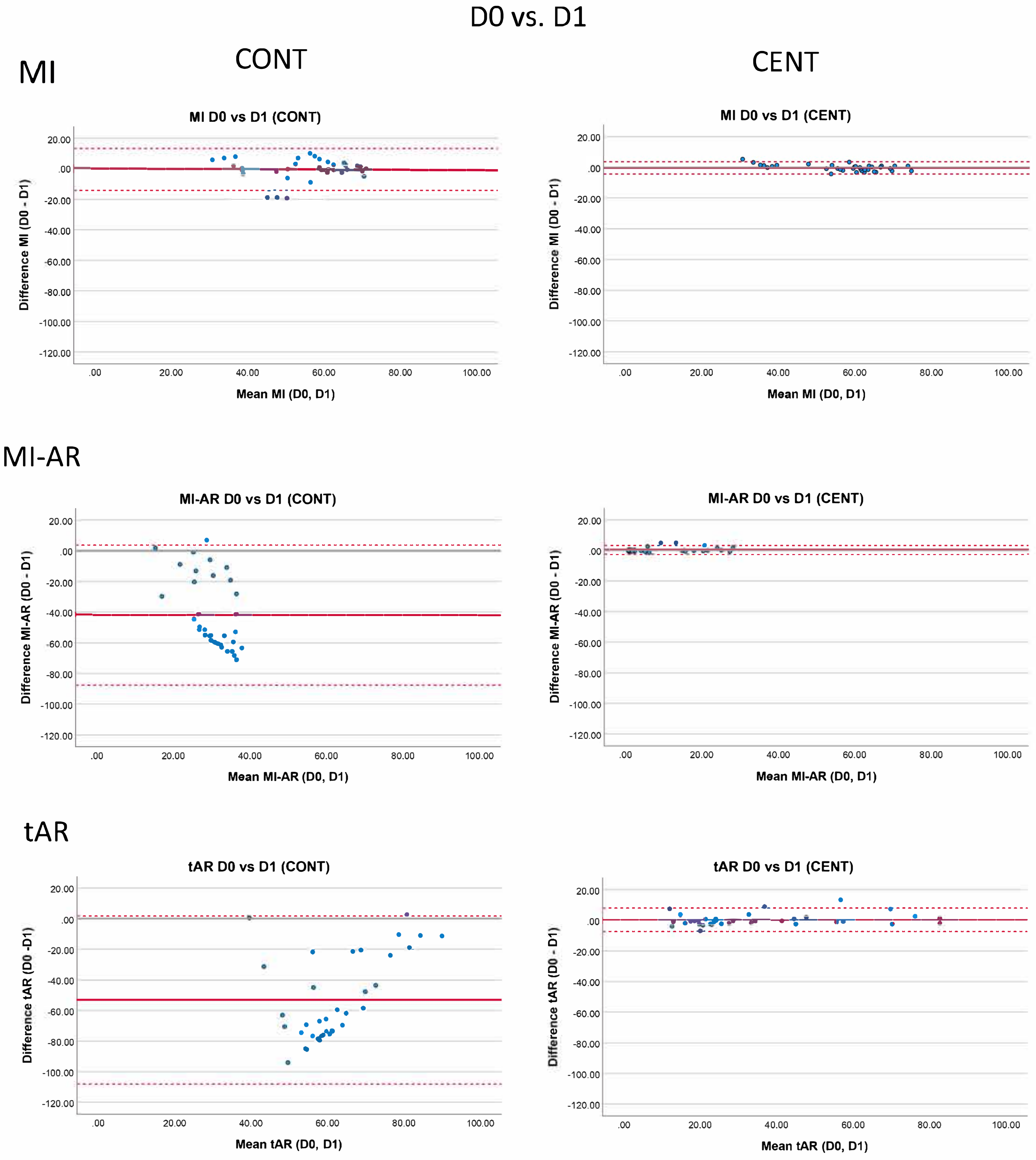

Supplementary Figure 2A: Bland-Altman plots showing the agreement in the analysis of the percentage of membrane intact (MI), membrane-intact acrosome-reacted (MI-AR) and total acrosome-reacted (tAR) sperm in samples exposed to vehicle or 10 µM A23187, between samples processed the same day they were stained (D0) or after storage for 24 h (D1). Samples were centrifuged to remove the staining either after (Control, CONT) or before (Centrifuged, CENT) storage. Each of the paired measures is represented by assigning the mean of the two values on the X axis and the difference between the two values on the Y axis. The solid red line represents the mean difference (bias), and the two dashed red lines the 95% limits of agreement (± 1.96 standard deviations from the mean difference).

Supplementary Figure 2B

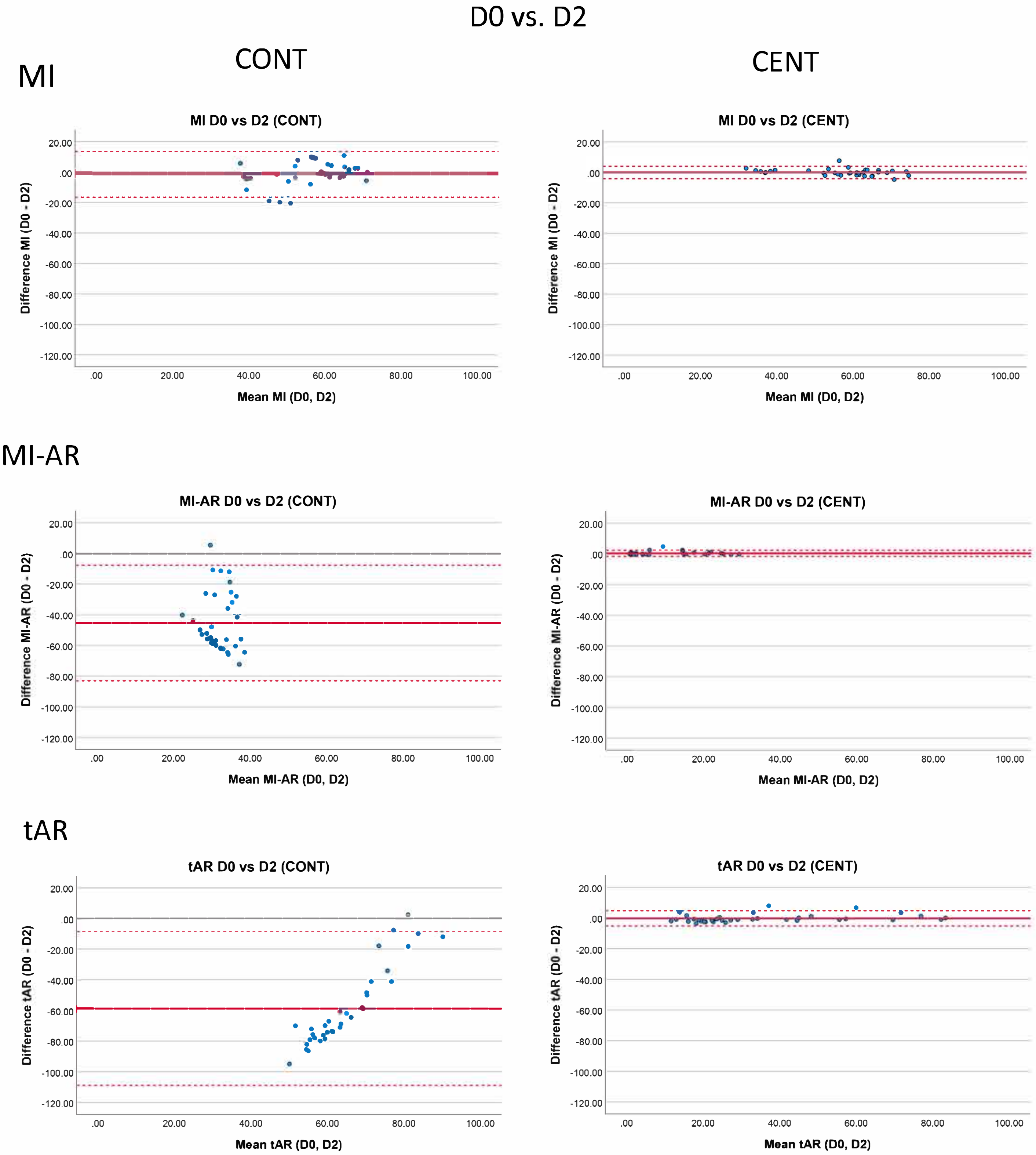

Supplementary Figure 2B: Bland-Altman plots showing the agreement in the analysis of the percentage of membrane intact (MI), membrane-intact acrosome-reacted (MI-AR) and total acrosome-reacted (tAR) sperm in samples exposed to vehicle or 10 µM A23187, between samples processed the same day they were stained (D0) or after storage for 48 h (D2). Samples were centrifuged to remove the staining either after (Control, CONT) or before (Centrifuged, CENT) storage. Each of the paired measures is represented by assigning the mean of the two values on the X axis and the difference between the two values on the Y axis. The solid red line represents the mean difference (bias), and the two dashed red lines the 95% limits of agreement (± 1.96 standard deviations from the mean difference).
